# *Klebsiella pneumoniae* DedA family proteins have redundant roles in divalent cation homeostasis and resistance to phagocytosis

**DOI:** 10.1128/spectrum.03807-23

**Published:** 2024-01-12

**Authors:** Vijay Tiwari, Amit Sharma, Reygan Braga, Emily Garcia, Ridhwana Appiah, Renee Fleeman, Basel H. Abuaita, Marianna Patrauchan, William T. Doerrler

**Affiliations:** 1 Department of Biological Sciences, Louisiana State University, Baton Rouge, Louisiana, USA; 2 Department of Pathobiological Sciences, LSU School of Veterinary Medicine, Louisiana State University, Baton Rouge, Louisiana, USA; 3 Department of Microbiology and Molecular Genetics, College of Arts and Science, Oklahoma State University, Stillwater, Oklahoma, USA; 4 Burnett School of Biomedical Sciences, College of Medicine, University of Central Florida, Orlando, Florida, USA; University of North Dakota, Grand Forks, North Dakota, USA

**Keywords:** divalent cations, membrane transport, capsular polysaccharide, phagocytosis

## Abstract

The DedA superfamily is a highly conserved family of membrane proteins. Deletion of *Escherichia coli yqjA* and *yghB*, encoding related DedA family proteins, results in sensitivity to elevated temperature, antibiotics, and alkaline pH. The human pathogen *Klebsiella pneumoniae* possesses genes encoding DedA family proteins with >90% amino acid identity to *E. coli* YqjA and YghB. We hypothesized that the deletion of *K. pneumoniae yqjA* and *yghB* will impact its physiology and may reduce its virulence. The *K. pneumoniae* Δ*yqjA* Δ*yghB* mutant (strain VT101) displayed a growth defect at 42°C and alkaline pH sensitivity, not unlike its *E. coli* counterpart. However, VT101 retained mostly wild-type resistance to antibiotics. We found VT101 was sensitive to the chelating agent EDTA, the anionic detergent SDS, and agents capable of alkalizing the bacterial cytoplasm such as bicarbonate or chloroquine. We could restore growth at alkaline pH and at elevated temperature by addition of 0.5–2 mM Ca^2+^ or Mg^2+^ to the culture media. VT101 displayed a slower uptake of calcium, which was dependent upon calcium channel activity. VT201, with similar deletions as VT101 but derived from a virulent *K. pneumoniae* strain, was highly susceptible to phagocytosis by alveolar macrophages and displayed a defect in the production of capsule. These findings suggest divalent cation homeostasis and virulence are interlinked by common functions of the DedA family.

**IMPORTANCE:**

*Klebsiella pneumoniae* is a dangerous human pathogen. The DedA protein family is found in all bacteria and is a membrane transporter often required for virulence and antibiotic resistance. *K. pneumoniae* possesses homologs of *E. coli* YqjA and YghB, with 60% amino acid identity and redundant functions, which we have previously shown to be required for tolerance to biocides and alkaline pH. A *K. pneumoniae* strain lacking *yqjA* and *yghB* was found to be sensitive to alkaline pH, elevated temperature, and EDTA/SDS and displayed a defect in calcium uptake. Sensitivity to these conditions was reversed by addition of calcium or magnesium to the growth medium. Introduction of Δ*yqjA* and Δ*yghB* mutations into virulent *K. pneumoniae* resulted in the loss of capsule, increased phagocytosis by macrophages, and a partial loss of virulence. These results show that targeting the *Klebsiella* DedA family results in impaired divalent cation transport and, in turn, loss of virulence.

## INTRODUCTION

*Klebsiella pneumoniae* is a gram-negative, encapsulated, opportunistic pathogen and a common cause of nosocomial infections including pneumonia, urinary tract infections, and wound infections (1–3). *K. pneumoniae* is also responsible for causing community-acquired infections such as endophthalmitis, pneumonia, meningitis, and pyogenic liver abscess in healthy individuals (4). Following initial attachment, *K. pneumoniae* can enter the bloodstream causing bacteremia, turning the infection into a life-threatening condition by displaying a high degree of virulence and antibiotic resistance (5–8). Due to the lack of effective antibiotics against *K. pneumoniae,* challenges in the treatment and management of its infections have become a major public health problem (9). Colistin monotherapy, or in combination with other antibiotics, has become a last resort treatment for multidrug resistant (MDR) *K. pneumoniae* (10). The major virulence factors of *K. pneumoniae* are capsule, lipopolysaccharides, siderophores, and pili (3, 11–13).

The DedA superfamily is a conserved family of integral membrane proteins with diverse roles in membrane transport. Previous studies have shown DedA family proteins play central roles in the maintenance of membrane potential, but the mechanism behind this is not fully understood (14–18). The *E. coli* genome encodes eight members of the DedA family (19). Deletion of two of these, YghB and YqjA, with 61% amino acid identity and partially redundant functions, causes sensitivity to antibiotics and elevated temperatures, induction of envelope stress responses, and cell division defects (14, 20, 21). *E. coli* Δ*yqjA* is sensitive to alkaline pH indicating a loss of regulation of internal pH, a downstream effect of altered membrane potential (15). Chemical alteration of membrane potential can reproduce the altered membrane potential seen in Δ*dedA* mutants (17, 18). Our work has shown that we can sensitize bacteria to colistin or lower their virulence by simply altering their membrane potential and also suggests the DedA superfamily represents a target for pharmacological interference with virulence and drug resistance (17, 18, 22).

In this study, we determined the impact of mutation of *K. pneumoniae yqjA* and *yghB*, encoding conserved members of the DedA superfamily sharing 63% amino acid identity. We found VT101 (*K. pneumoniae* MKP103; Δ*yqjA*, Δ*yghB*) is sensitive to alkaline pH and elevated temperatures. The membrane of VT101 is hyperpolarized, but the strain displays wild-type resistance to most antibiotics. Instead, the strain is markedly sensitive to ethylenediaminetetraacetic acid (EDTA) and SDS. Most phenotypes can be reversed by the addition of divalent cations, calcium or magnesium, to the growth media, and we show the strain is compromised in calcium uptake. A Δ*yqjA*, Δ*yghB* strain derived from virulent *K. pneumoniae* ST258 (VT201) is compromised in capsule synthesis, is more susceptible to phagocytosis by murine alveolar macrophages (AMs), and is less virulent in the wax moth *Galleria mellonella*. These results show that members of the DedA superfamily contribute to divalent cation homeostasis, capsule synthesis, and virulence.

## RESULTS

### *In silico* analysis to identify *Klebsiella pneumoniae* YqjA and YghB

**Fig 1:**
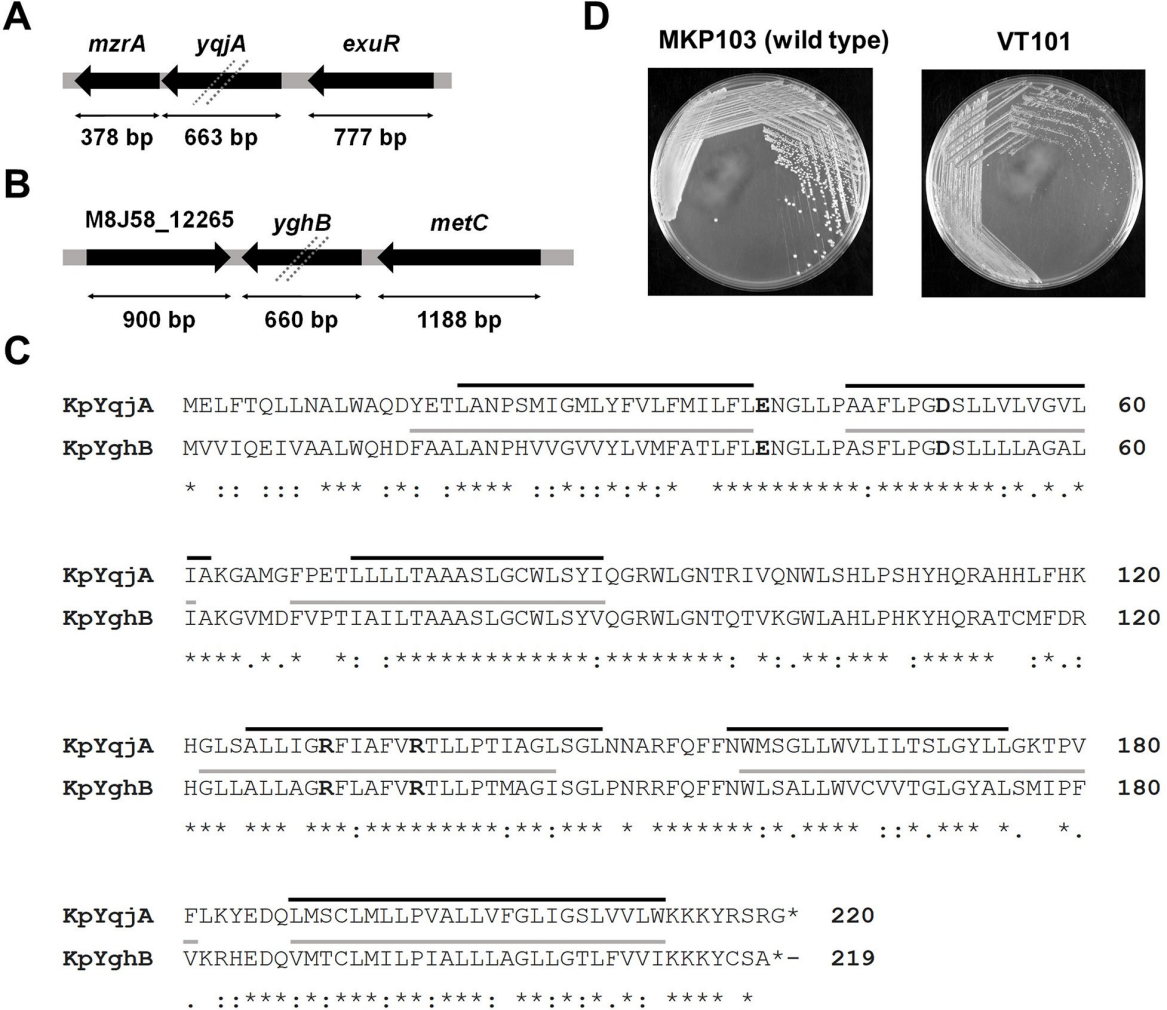
(A and B) Genomic map of *K. pneumoniae yqjA* (**A**) and *yghB* (**B**) showing their neighboring genes. Dashed lines indicate gene deletions in VT101 produced using λ red recombination (24). (**C**) Amino acid sequence alignment showing similarity between *K. pneumoniae* YqjA and YghB. Amino acid alignment was carried out using Clustal Omega (25). Acidic amino acids (E39, D51) and basic amino acids (R130, R136) in bold fonts, previously shown to be essential in *E. coli* YqjA and YghB (20, 26) are conserved in *K. pneumoniae* YqjA and YghB. Solid gray and black lines indicate predicted membrane domains using TMHMM (27). (**D**) VT101 displays a growth defect on Luria-Bertani plates compared to wild-type *K. pneumoniae* MKP103. Plates were incubated at 30°C for 24 hours.

We used *E. coli* MG1655 YqjA as the template to conduct a BLAST search against the *K. pneumoniae* KPNIH1 genome (accession # PRJNA73191) (23). We found the top three hits displayed significant identity and encoded DedA superfamily proteins. Amino acid sequence alignment between the first hit in the BLAST search and *E. coli* YqjA showed a 92.3% identity, and we assigned this as *Klebsiella* YqjA. Furthermore, amino acid sequence alignment between the second hit and *E. coli* YghB showed 90.4% identity, and we assigned the second hit as *Klebsiella* YghB. Identical or nearly identical homologs of YqjA and YghB are found in all sequenced *K. pneumoniae* genomes. The genomic vicinity for each gene is shown in Fig. 1A and B. *Klebsiella* YqjA and YghB display 63% amino acid identity (Fig. 1C). Expression of cloned *Klebsiella yqjA* or *yghB* in *E. coli dedA* family mutants restored growth at 42°C and at alkaline pH, demonstrating their functionality in the *E. coli* host (Fig. S1).

In addition to YqjA and YghB, *K. pneumoniae* encodes a third member of the DedA family, DedA itself. We reported previously that *K. pneumoniae* Δ*dedA* is sensitive to colistin and is defective in virulence in a *Galleria mellonella* model (18). This gene was named *dkcA* (*dedA* of *Klebsiella* required for colistin resistance). DkcA was not included in this study because it shows only limited amino acid identity to YqjA and YghB (~30%).

### Sensitivity of VT101 to alkaline pH and rescue by Ca^2+^ or Mg^2+^

Our previous work has shown a functional redundancy between *E. coli* YqjA and YghB. Simultaneous deletion of both genes in *E. coli* results in sensitivity to alkaline pH, elevated temperatures, and certain antibiotics and biocides (20, 21, 28). We hypothesized *Klebsiella* YqjA and YghB play similar roles. *K. pneumoniae* MKP103 with Δ*yqjA* and Δ*yghB* mutations was named VT101 and was routinely grown at 30°C on Luria-Bertani (LB) medium at pH 7.0, where it was found to form significantly smaller-sized colonies (Fig. 1D).

**Fig 2:**
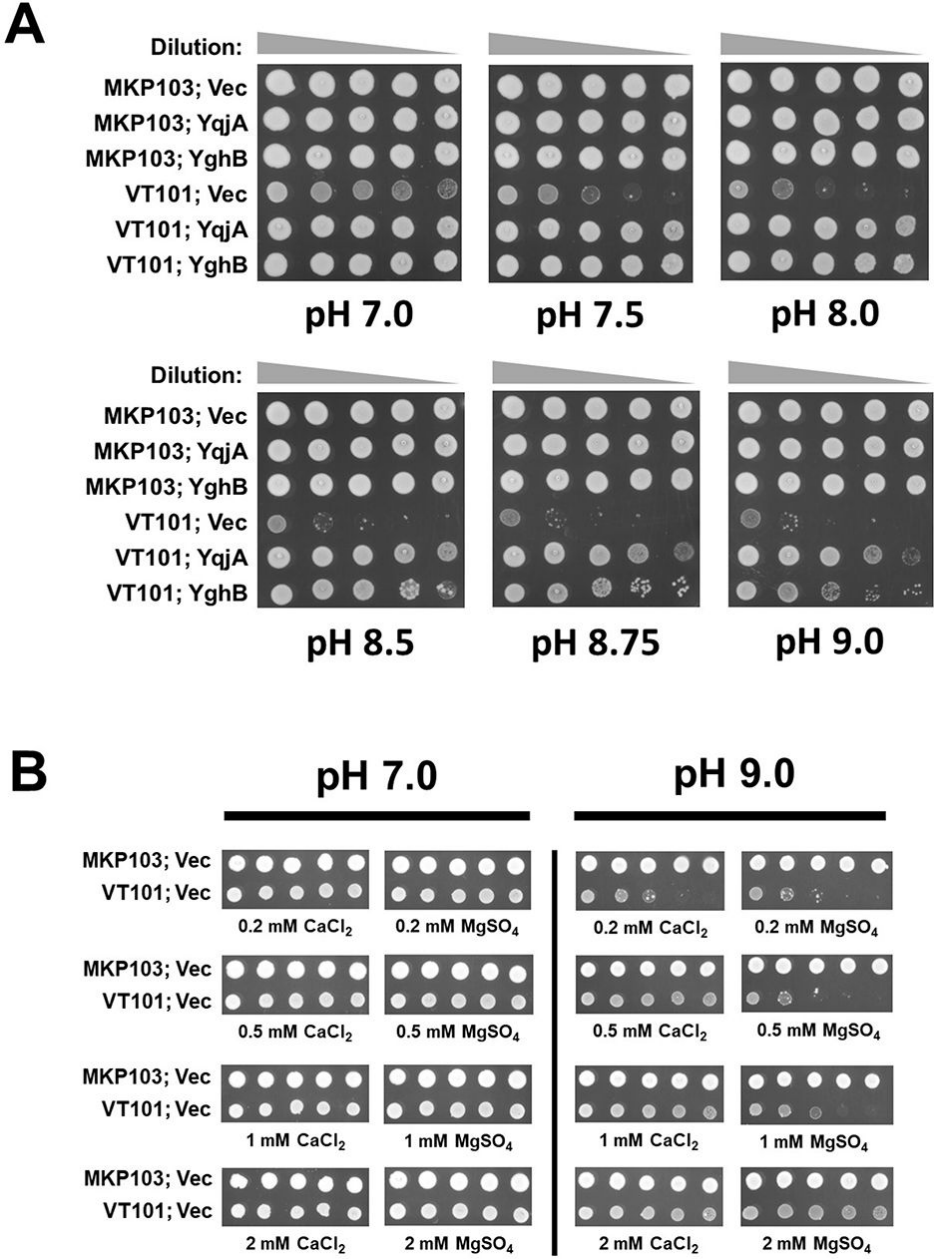
VT101 is sensitive to alkaline pH, and Ca^2+^ or Mg^2+^ can suppress alkaline pH sensitivity. (**A**) VT101 struggles to grow at alkaline pH. VT101 transformed with control vector (vec), pBAD (KpYqjA), or pBAD (KpYghB) was grown overnight, serially diluted, and spotted on LB plates containing 0.1% arabinose and 50 µg/mL apramycin. (**B**) Ca^2+^ and Mg^2+^ promote the survival of VT101 at alkaline pH. MKP103 and VT101 transformed with control vector (vec) were diluted from overnight culture and spotted on LB plates containing the indicated concentration of CaCl_2_ or MgSO_4_. The pH of the solid media was adjusted using NaOH. Plates were incubated at 30°C for 24 hours.

VT101 was as resistant as wild type to most antibiotics tested (Table 1). However, VT101 was sensitive to pH ≥7.5, and this sensitivity could be genetically complemented by the expression of either wild-type *K. pneumoniae yqjA* or *yghB* behind an arabinose-inducible promoter (Fig. 2A), highlighting the functional redundancy of these two DedA family proteins. We were also able to rescue alkaline pH sensitivity of VT101 by addition of 0.5 mM Ca^2+^ or 2 mM Mg^2+^ to the growth media (Fig. 2B), suggesting a possible alteration of divalent cation homeostasis in the mutant.

**TABLE 1:** Minimal inhibitory concentration (µg/mL) of select antimicrobial agents^*b*^

Strain	Nal	Col	Amp	Novo	Bac	Vanco	Tet	Erythro	Gent	EDTA
MKP103	>250	500	5,000	1,000	>1,000	>1,000	31.25	>1,000	>5,000	25^*a*^
VT101	>250	500	2,500	250	>1,000	>1,000	31.25	>1,000	>5,000	1.56^*a*^
ST258	>250	125	>5,000	125	>1,000	>1,000	<1.95	>1,000	31	62.5
VT201	>250	125	>5,000	62.5	>1,000	>1,000	3.91	>1,000	250	62.5

^*a*^ EDTA MIC measured in millimolar.

^*b*^ Nal, nalidixic acid; Col, colistin; Amp, ampicillin; Novo, novobiocin; Bac, bacitracin; Vanco, vancomycin; Tet, tetracycline; Erythro, erythromycin; Gent, gentamicin; nd, not determined.

### Sensitivity of VT101 to chemical agents alkalizing cytoplasmic pH and rescue by Ca^2+^ or Mg^2+^

**Fig 3:**
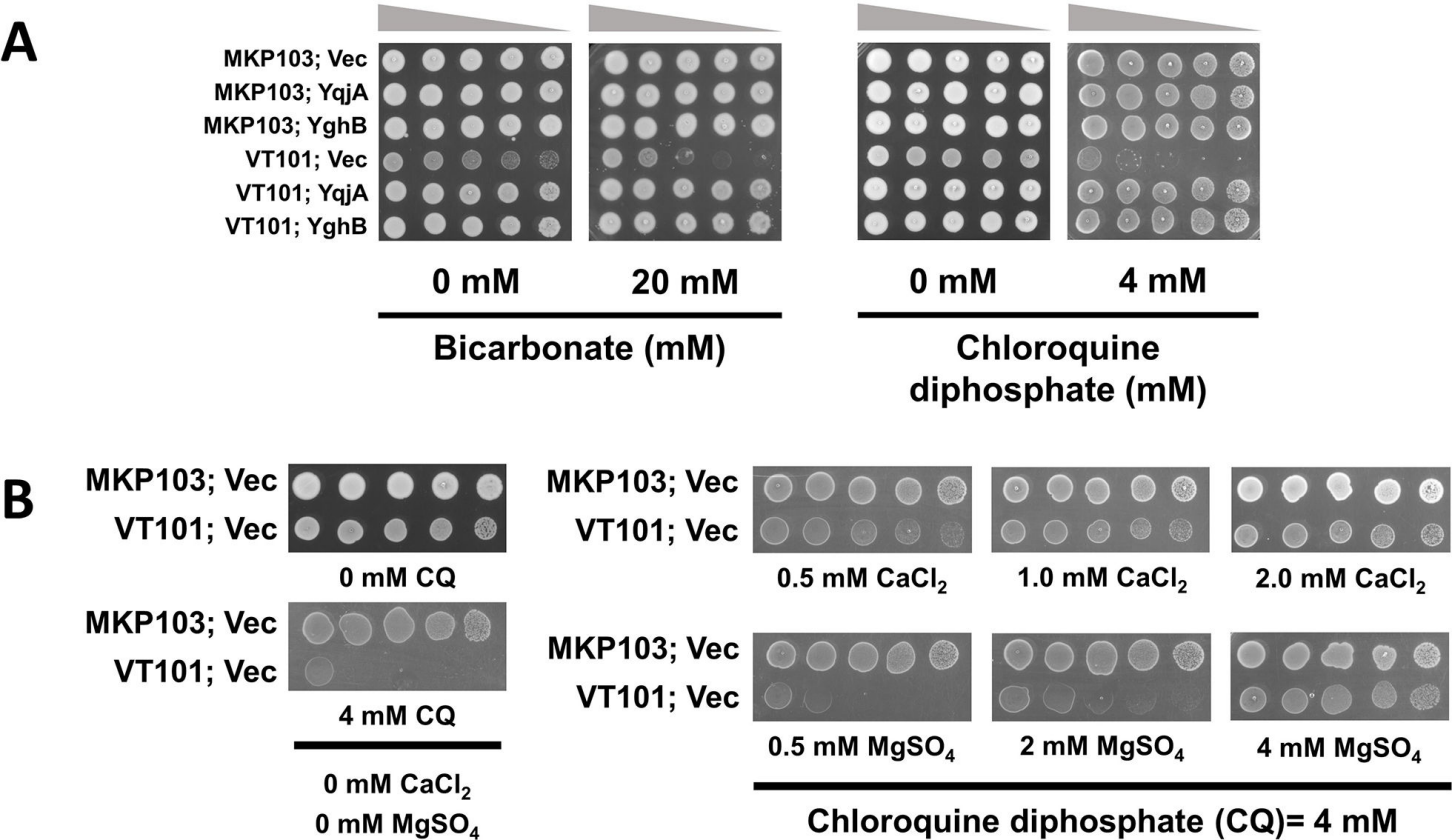
VT101 is sensitive to chemical agents causing alkalization of the cytoplasm, and Ca^2+^ or Mg^2+^ can rescue this sensitivity. (**A**) VT101 struggles to grow in the presence of alkalizing agents. VT101 transformed with control vector (vec), pBAD (KpYqjA), or pBAD (KpYghB) was grown overnight, serially diluted, and spotted on LB plates containing the indicated concentration of sodium bicarbonate or CQ, 0.1% arabinose, and 50 µg/mL apramycin. (**B**) Ca^2+^ is more efficient in suppressing the effect of CQ compared to Mg^2+^. MKP103 and VT101 transformed with control vector (vec) were diluted from overnight culture and spotted on LB plates containing 4 mM CQ and the indicated concentration of CaCl_2_ or MgSO_4_. Single control plates containing LB or LB/CQ from the same internally controlled experiment are shown. Plates were incubated at 30°C for 24 hours.

Sodium bicarbonate (NaHCO_3_) and chloroquine diphosphate salt (CQ) alkalize the cytoplasmic pH in bacterial cells (29, 30). VT101 was sensitive to 20 mM NaHCO_3_ and 4 mM CQ (Fig. 3A). Sensitivity to NaHCO_3_ and CQ was corrected by the expression of wild-type *yqjA* or *yghB* from a plasmid (Fig. 3A). We were also able to restore the resistance of VT101 to CQ by adding 0.5 mM Ca^2+^ or 3–4 mM Mg^2+^ to the media (Fig. 3B). We concluded VT101 is sensitive to chemical agents alkalizing the cytoplasm, and *Klebsiella* YqjA and YghB display functional redundancy to complement this sensitivity. Divalent cations (Ca^2+^ or Mg^2+^) also alleviate the sensitivity of VT101 toward alkalizing agents.

### Sensitivity of VT101 to elevated temperature and rescue by Ca^2+^ or Mg^2+^

**Fig 4:**
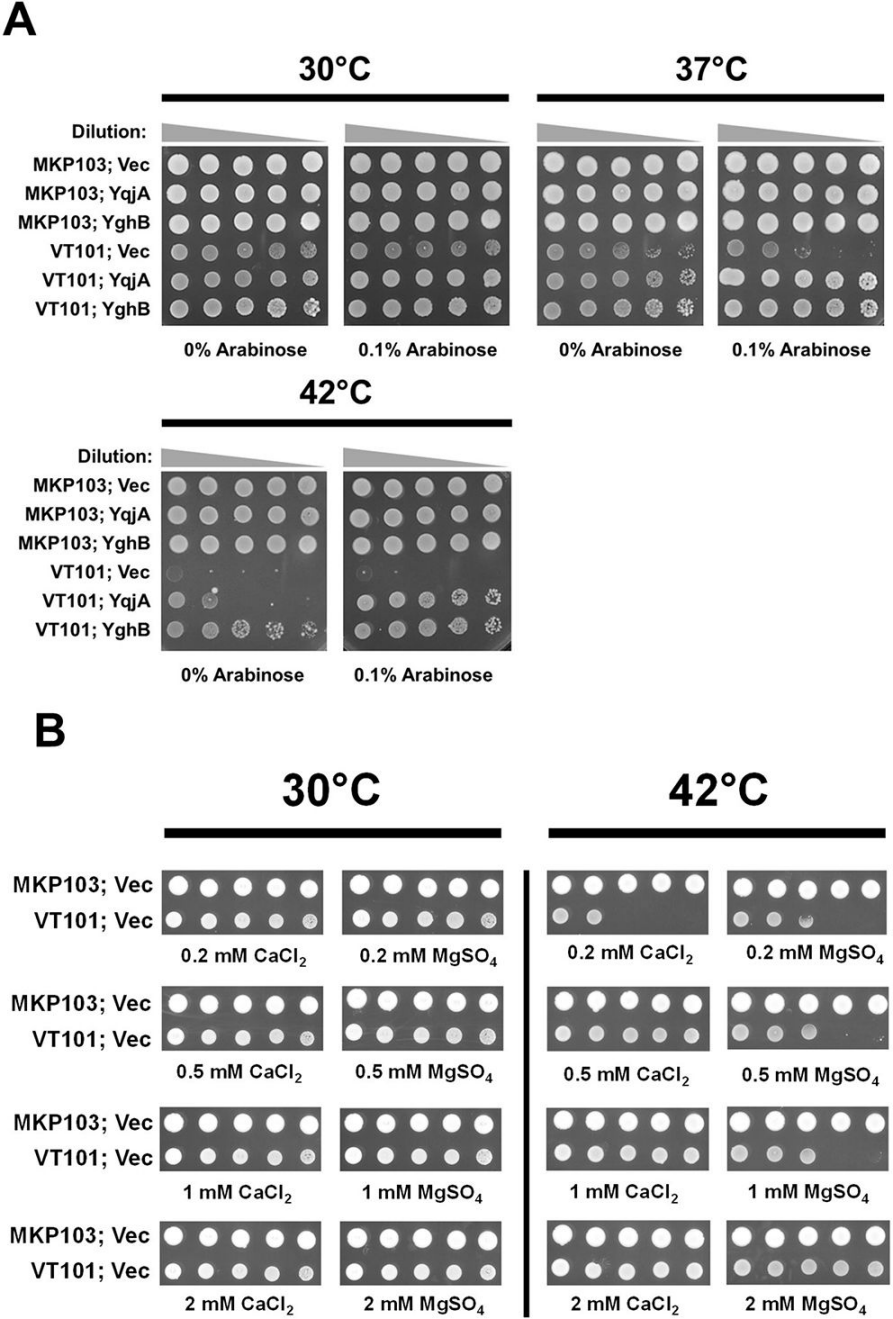
VT101 shows growth sensitivity to elevated temperature, and Ca^2+^ or Mg^2+^ can rescue this sensitivity. (**A**) VT101 struggles to grow at 42°C. VT101 was transformed with control vector (vec), pBAD (KpYqjA) or pBAD (KpYghB) was grown overnight, serially diluted, and spotted on LB plates containing 0.1% arabinose and 50 µg/mL apramycin. (**B**) Ca^2+^ or Mg^2+^ can restore the growth of VT101 at 42°C. MKP103 and VT101 transformed with control vector (vec) were diluted from overnight culture and spotted on LB plates with indicated concentrations of CaCl_2_ or MgSO_4_. Plates were incubated at 30°C and 42°C for 24 hours.

*E. coli* Δ*yqjA*, Δ*yghB* displays sensitivity to elevated temperatures (28). To test the temperature sensitivity of VT101, we spotted dilutions of the strains at three different temperatures (30°C, 37°C, and 42°C). VT101 shows a growth defect at 37°C and completely ceased to grow at 42°C (Fig. 4A). Growth sensitivity of VT101 at elevated temperature was rescued by expression of *Klebsiella yqjA* or *yghB* (Fig. 4A). In *E. coli*, expression of Mg^2+^ transport channels such as CorA is induced at elevated temperatures (31), and in *Salmonella*, a mutation causing constitutive expression of Mg^2+^ transporter MgtA results in enhanced thermotolerance (32). Each of these findings is consistent with an important role for Mg^2+^ in the survival of bacteria at high temperature. Ca^2+^ as low as 0.5 mM and Mg^2+^ as low as 2 mM were able to correct the temperature sensitivity of VT101 (Fig. 4B). We hypothesized that sensitivity of VT101 to high temperature could be a result of disruption in divalent cation transport (Ca^2+^ or Mg^2+^).

### VT101 is highly sensitive to EDTA and SDS

**Fig 5:**
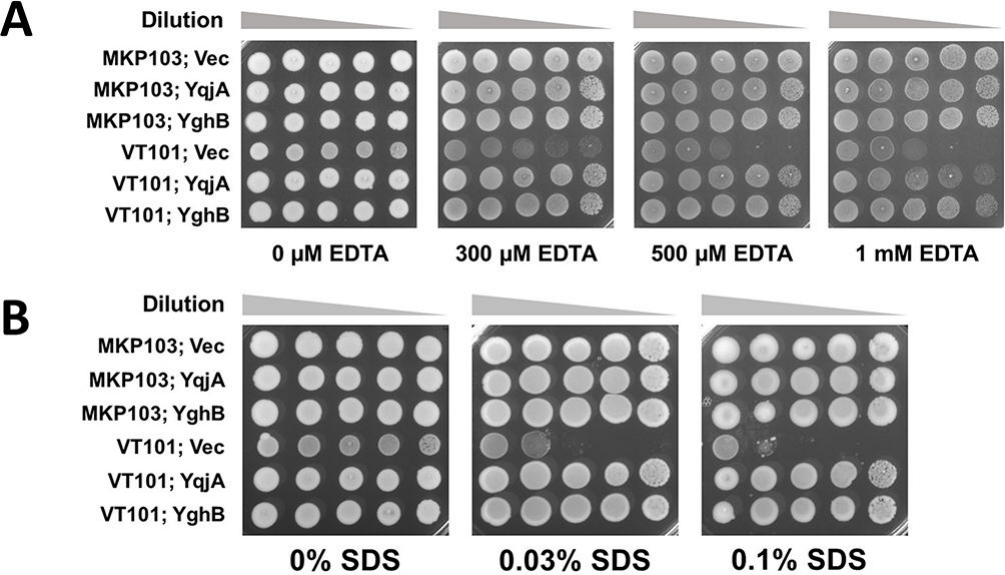
*K. pneumoniae* VT101 fails to grow in the presence of EDTA or SDS. (**A**) Sensitivity of VT101 to EDTA. Dilutions of indicated strains from overnight cultures were spotted and grown on LB plates with indicated concentration of EDTA, 0.1% arabinose, and 50 µg/mL apramycin. (**B**) Sensitivity of VT101 to SDS. Dilutions of indicated strains from overnight cultures were spotted and grown on LB plates with indicated concentration of SDS, 0.1% arabinose, and 50 µg/mL apramycin. Plates were incubated at 30°C for 36 hours.

Supplementing the growth media with divalent cations (Ca^2+^ or Mg^2+^) in the range of 0.5–2 mM supported the growth of VT101 under stressors such as high alkaline pH, cytoplasmic alkalinization, or high temperature. This suggests VT101 struggles to maintain divalent cation homeostasis. We hypothesized exposure of VT101 to EDTA (a Ca^2+^ and Mg^2+^ chelator) will further exacerbate the altered divalent cation homeostasis in VT101. Using broth microdilution, we found the EDTA MIC for wild type, and VT101 was 25 and 1.56 mM, respectively (Table 1). VT101 sensitivity toward EDTA was restored to wild-type levels by expression of *Klebsiella yqjA* or *yghB* from a plasmid (Fig. 5A). In conclusion, EDTA imparts a lethal effect on VT101 cells that are already struggling to cope with altered divalent cation homeostasis. EGTA had a similar effect (data not shown).

Ca^2+^ and Mg^2+^ take part in electrostatic cross-links with negatively charged phosphate groups between adjacent LPS molecules (33). Bacteria lose significant amount of LPS if electrostatic cross-linking with divalent cations is disrupted using chelating agents such as EDTA (34). Since we did not detect gross changes in total amounts of LPS or LPS structure using silver staining (Fig. S2) and we did not see changes in sensitivity to vancomycin (Table 1) suggesting an intact OM (35), the sensitivity to SDS may be explained by changes in LPS organization in the OM. To test this, we performed a spotting assay on LB plates with different concentrations of SDS. VT101 struggled to grow on LB with 0.03% SDS and was completely inhibited by 0.1% SDS, while the parent strain grew in the presence of all tested concentrations (Fig. 5B). Expression of *Klebsiella yqjA* or *yghB* was sufficient to render VT101 resistant to SDS. This finding suggests a loss of OM integrity in VT101 is linked to alterations of divalent cation transport.

### Membrane hyperpolarization of VT101

**Fig 6:**
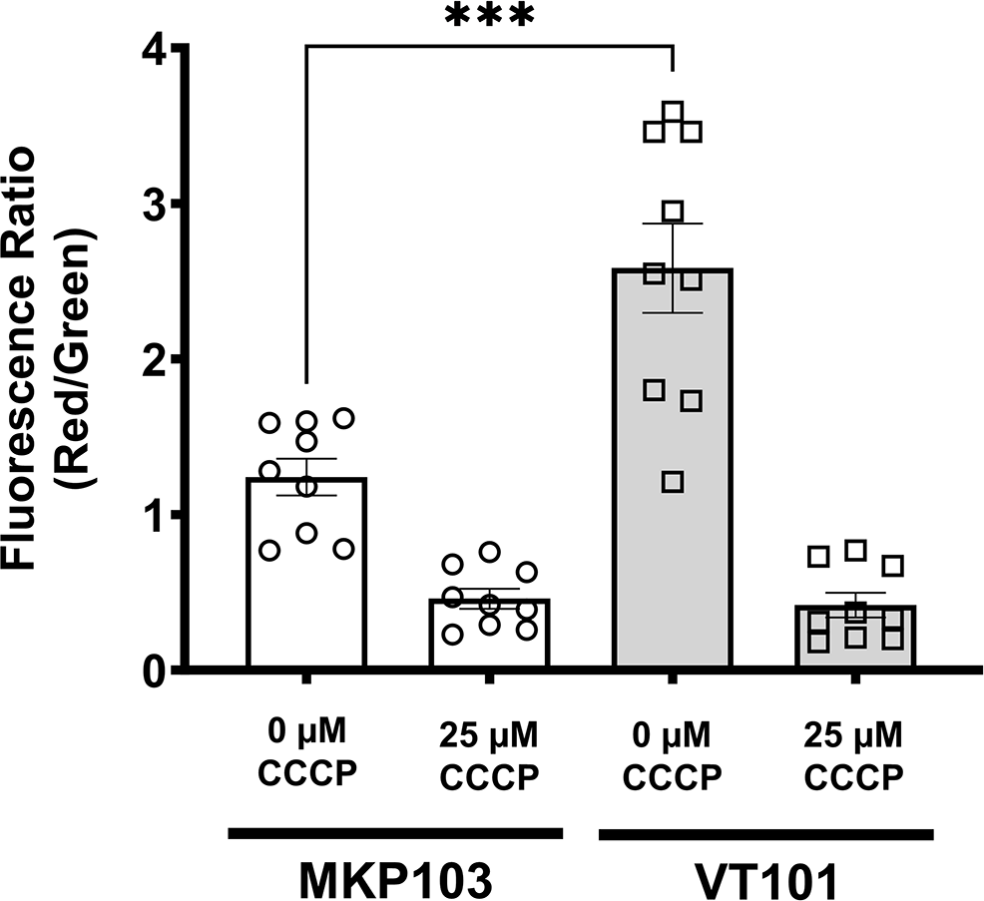
*K. pneumoniae* VT101 displays significant hyperpolarization of the membrane potential (ΔΨ). Treatment of MKP103 and VT101 with 25 µM CCCP for 30 min resulted in the loss of ΔΨ was used as the control. Bars represent mean ± SEM of nine biological replicates. The experiment was repeated three times. The Mann-Whitney test was used to perform statistical analysis. Statistical comparisons were considered significant at *P* < 0.05. ***, *P* < 0.001.

The proton motive force (PMF) is defined as the sum of the electrical gradient (ΔΨ) and pH gradient (ΔpH) across a membrane. Most neutralophiles maintain a constant PMF across a wide range of external environments by altering one or both components of the PMF (36). *K. pneumoniae* and *B. glumae dedA* family mutants showed significant membrane hyperpolarization (ΔΨ), linked to colistin sensitivity and loss of virulence (17, 18). Since the phenotypes of VT101 suggested altered membrane transport, we measured ΔΨ of *K. pneumoniae* wild type and VT101 using the JC-1 dye. JC-1 is a lipophilic, fluorescent cationic dye, exhibiting green fluorescence (~530 nm) at its natural monomeric form. In a healthy bacterial cell, JC-1 forms aggregates in a concentration-dependent manner called J aggregates, shifting its fluorescence emission from green to red (~590 nm) (37). The red:green ratio for *K. pneumoniae* wild type was 1.24 ± 0.12, whereas red:green ratio for VT101 was 2.58 ± 0.29, twofold higher for VT101 than the ratio observed for wild type (Fig. 6). Treatment of wild type and VT101 with 25 µm protonophore *m*-chlorophenyl carbonyl cyanide hydrazone (CCCP) caused dissipation of ΔΨ. We concluded VT101 is hyperpolarized compared to the wild type. While we were unable to consistently measure the ΔpH component of the PMF, changes in ΔΨ reflect altered ΔpH in order to maintain the PMF at a constant level (36). As we reported previously, we found inducer arabinose influences the ΔΨ of *K. pneumoniae* (18). Therefore, we were unable to measure the effect of genetic complementation on membrane polarization.

### VT101 has slower uptake of Ca^2+^

Bacteria can acquire divalent cations (Ca^2+^ or Mg^2+^) constitutively using ion channels or actively using P-type ATPases (38, 39). While many questions remain about the exact mechanisms used to maintain calcium homeostasis (40), it is known that the low cytoplasmic calcium concentration relative to the external concentration is maintained primarily by active efflux by P-type ATPases and antiporters and influx by Ca channels (41). Since depolarization is known to stimulate ion channel activity (42), we reasoned that hyperpolarization may inhibit channel activity. To test this hypothesis, we measured Ca^2+^ uptake in wild type and VT101 using the calcium-activated reporter protein aequorin cloned into an inducible plasmid (43).

**Fig 7:**
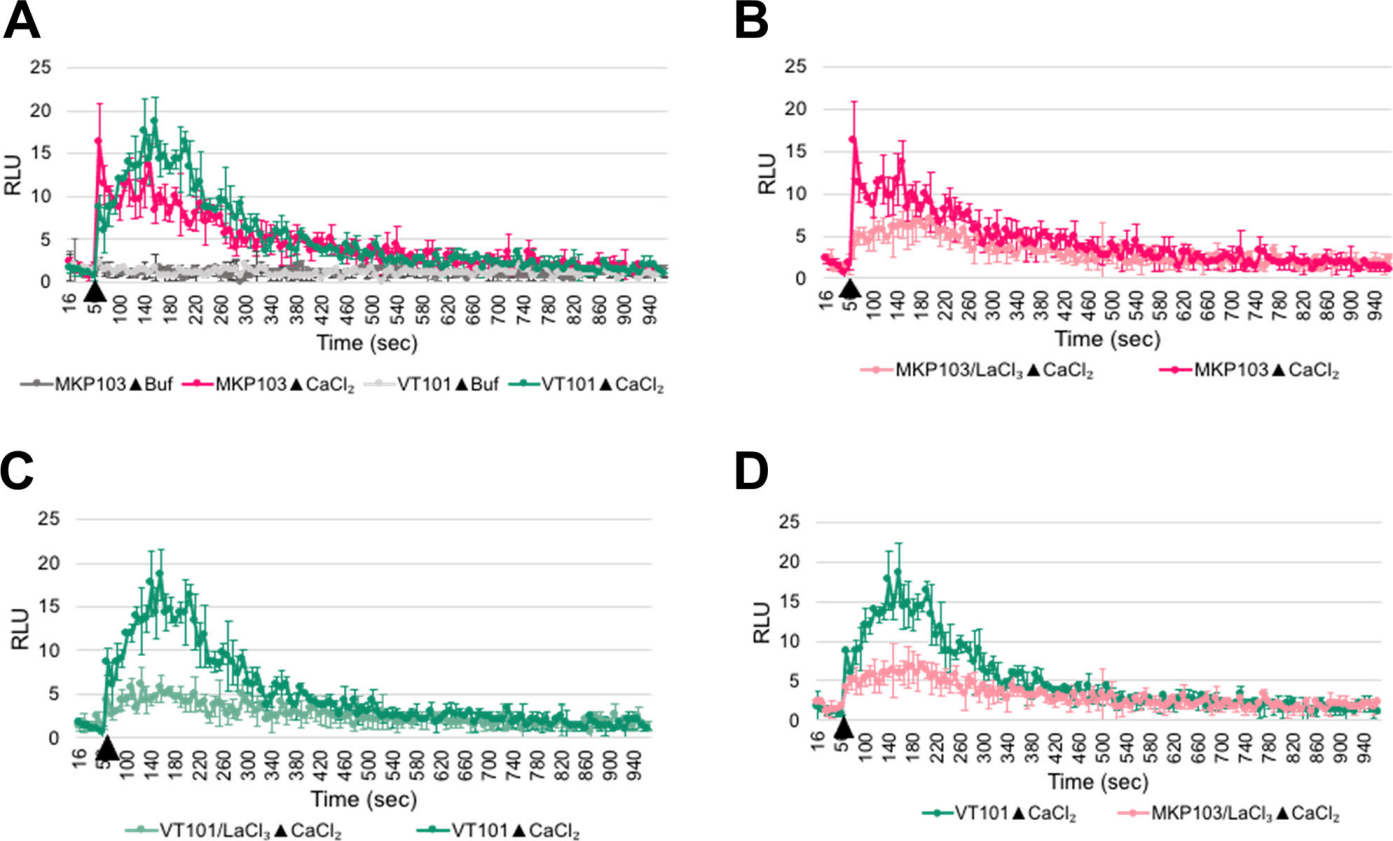
*K. pneumoniae* VT101 shows a slower uptake of Ca^2+^. (**A**) Calcium uptake was measured in MKP103 and VT101 after challenging with 1 mM CaCl_2_. (B and C) MKP103 and VT101 were treated with 600 µM lanthanum chloride (LaCl_3_), calcium channel blocker, before challenging the cells with 1 mM CaCl_2_. (**D**) Comparing the intracellular calcium between wild type treated with LaCl_3_ and VT101. The (apo)aequorin expression was induced for 8 hours using 0.5% arabinose. After reconstitution of aequorin using 5 µM coelenterazine, cells were challenged with a final concentration of 1 mM CaCl_2_ to study Ca^2+^ homeostasis. In both wild type and VT101, 1 mM CaCl_2_ was introduced at 60 s time point.

Since intracellular Ca^2+^ homeostasis in *K. pneumoniae* has not been characterized, we first aimed to establish the abilities of the organism to maintain basal level of intracellular free Ca^2+^ and to respond to exogenous Ca^2+^. For this, we monitored the basal level of Ca^2+^ for 60 s and then followed the changes in the intracellular free Ca^2+^ upon injection of 1 mM CaCl_2_. As expected, the luminescence level in resting cells remained low. After the introduction of CaCl_2_, the luminescence spiked rapidly in the wild-type cells and reached its peak value of 16.3 ± 4.5 RLU at 68 s (Fig. 7A). We concluded wild-type cells were able to maintain the basal level of Ca^2+^ and had robust uptake of exogenous Ca^2+^. In contrast, the luminescence increased more gradually in VT101 cells after the introduction of 1 mM CaCl_2_ and peaked at 18.7 ± 2.9 RLU at 156 s (Fig. 7A). We concluded the calcium uptake was significantly slower in VT101 compared to wild type. Although with slight difference in the rate, both wild-type and mutant cells were able to return the luminescence back to the basal level at 540 s, suggesting (i) the mutation had a minor impact on Ca^2+^ efflux, and (ii) the presence of Ca^2+^ efflux mechanisms that were not affected by the mutation.

To test the involvement of Ca^2+^ channel activity in *K. pneumoniae* Ca^2+^ uptake, both wild type and VT101 were treated with 600 µM lanthanum chloride (LaCl_3_), a Ca^2+^ channel antagonist (Fig. 7B and C). The treatment significantly reduced the uptake of Ca^2+^ in both the wild type and VT101. This suggests that *K. pneumoniae* possess Ca^2+^ channel activity that is responsible for the uptake of exogenous Ca^2+^ and indicates that the slower rate of Ca^2+^ uptake in the mutant (Fig. 7A) is likely due to alteration in this activity. Since the intracellular Ca^2+^ in wild type after LaCl_3_ treatment was significantly lower than that of untreated VT101 (Fig. 7D), the mutation only partially impaired Ca^2+^ channel activity. Overall, the data support a model whereby *Klebsiella* YqjA and YghB play roles in maintaining membrane potential, ensuring a perpetual driving force for Ca^2+^ channels, and thus contribute to proper Ca^2+^ homeostasis.

### VT201 displays a defect in polysaccharide capsule formation

**Fig 8:**
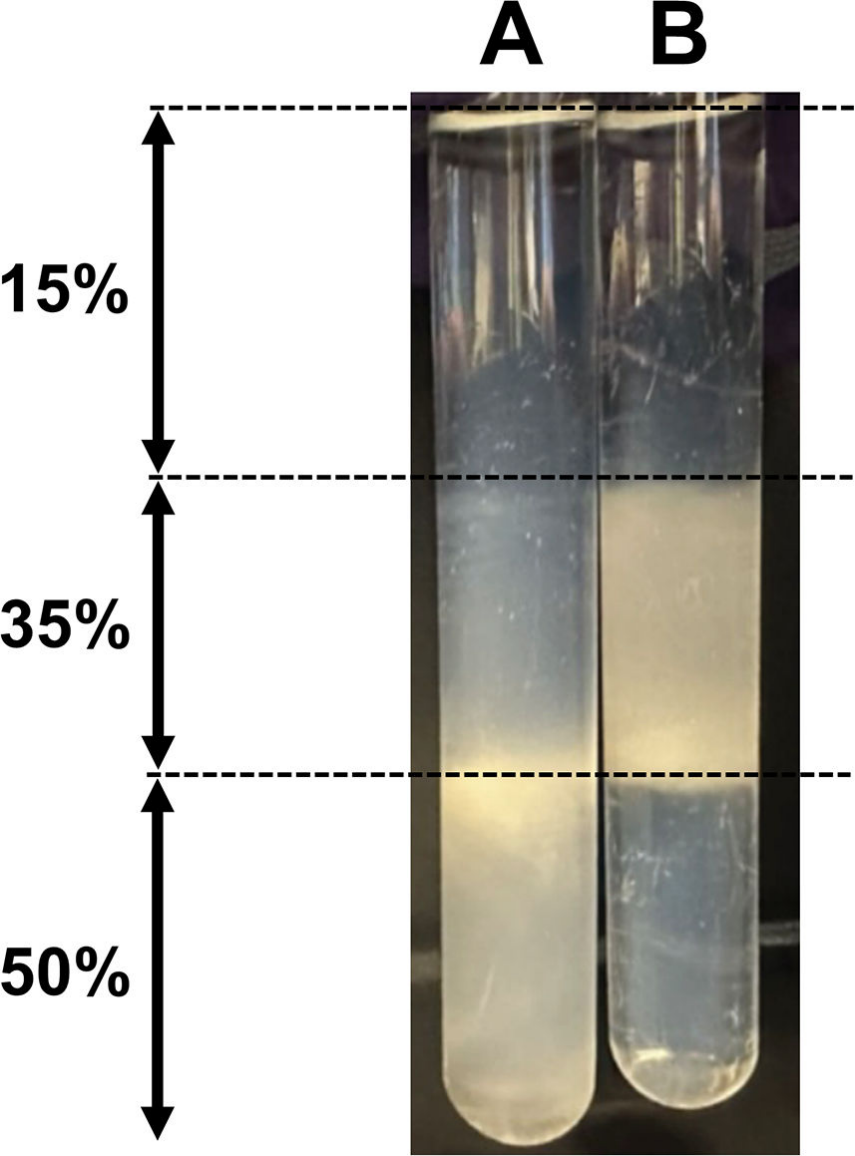
*K. pneumoniae* VT201 possesses less capsule than wild type. To measure the capsular content of VT201 (**A**) and wild-type ST258 (**B**), a semiquantitative Percoll gradient assay was used with low-speed centrifugation on a three-layer gradient of 15%, 35%, and 50%. A representative gradient is shown. The experiment was repeated three times.

We were interested in determining if *K. pneumonia yghB* and *yqjA* are required for virulence. To address this, we introduced the Δ*yqjA* and Δ*yghB* mutations into the virulent strain ST258 (44) to produce strain VT201. Capsule synthesis is a key feature of many virulent bacterial strains (45). The first step of capsule biosynthesis in *K. pneumoniae* involves the linking of hexoses to universal lipid carrier, undecaprenyl phosphate (Und-P) by inner membrane glycosyltransferases (45). Recent studies reported the identification of DedA family proteins in a screen for potential recycling flippases of Und-P (46, 47). We hypothesized Und-P recycling is impaired in VT201 compromising its capsule biosynthesis. We used a previously published protocol to semiquantitatively estimate capsule content in wild-type *K. pneumoniae* ST258 and VT201 (48). In this assay, we used a three-layer discontinuous Percoll gradient (15%, 35%, and 50%) followed by centrifugation (Fig. 8). VT201 was mainly found in the 50% gradient layer (Fig. 8A), while the parent strain was most abundant at the 35% gradient layer (Fig. 8B). Based on these findings, we conclude VT201 has significantly lower capsule content, possibly due to limiting amounts of Und-P available for capsule synthesis. VT101 displayed a similar phenotype (data not shown).

### VT201 is phagocytosed in higher numbers by alveolar macrophages

**Fig 9:**
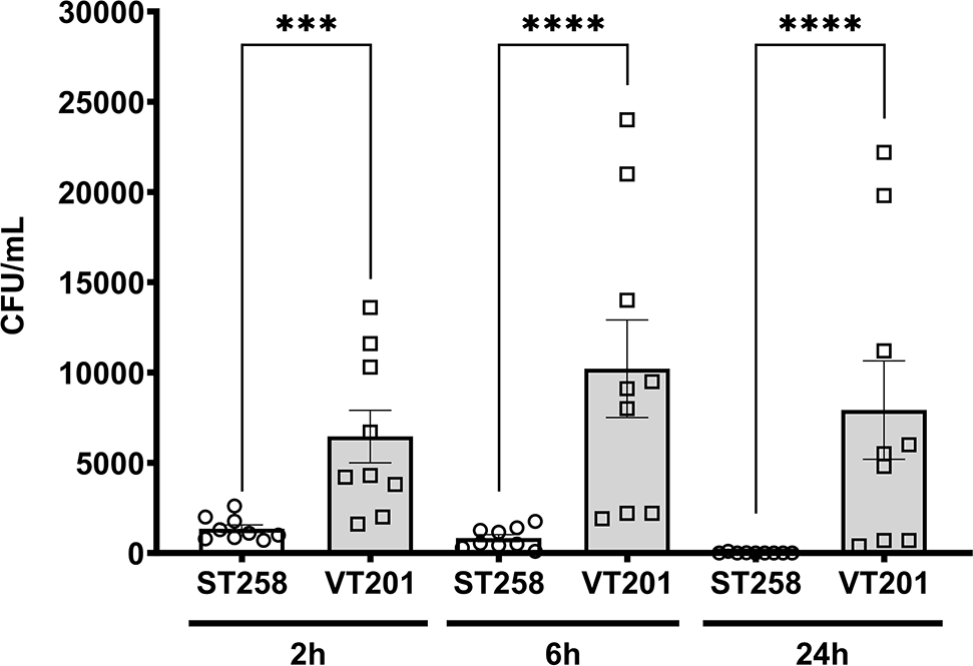
YqjA and YghB are required to resist phagocytosis by murine alveolar macrophage cells (MH-S). VT201 was phagocytosed in significantly higher numbers compared to wild-type ST258. Overnight cultures of wild type and VT201 were adjusted to OD600 = 1.0 and used to infect the AMs (MH-S) with multiplicity of infection of 1:10. Infected MH-S cells were incubated at 37°C with 5% CO_2_. Bacterial cells were harvested at selected time points by lysing MH-S cells, and serial dilution was carried out to estimate the CFU. Bars represent mean ± SEM of nine biological replicates. The experiment was repeated three times. The Mann-Whitney test was used to perform statistical analysis. Statistical comparisons were considered significant at *P* < 0.05. ***, *P* < 0.001; ****, *P* < 0.0001.

Capsular polysaccharide helps *K. pneumoniae* to escape phagocytosis from macrophages by protecting bacteria from opsonization (49, 50). We hypothesized VT201 may be phagocytosed more readily by murine alveolar macrophages due to less capsular polysaccharides. Following *in vitro* infection of AMs, we harvested bacterial cells after lysing macrophages at 2, 6, and 24 hours. For all selected time points, we saw a significantly higher number of VT201 compared to wild type (Fig. 9).

VT201 was unexpectedly more resistant to gentamicin than ST258 (Table 1). Therefore, we used a relatively high concentration (300 µg/mL) of the antibiotic to remove extracellular bacteria. For this reason, a control experiment was carried out to rule out gentamicin seepage into the AMs (Fig. S3). The numbers of intracellular bacteria remained largely unchanged at 6 hours post-infection even if we reduced the time of exposure to gentamicin to 30 min. We concluded significantly higher numbers of VT201 are phagocytosed by the AM cells due to less encapsulation. However, VT201 remains capable of intracellular survival based on our findings.

### VT201 displays a defect in virulence in *Galleria mellonella*

**Fig 10:**
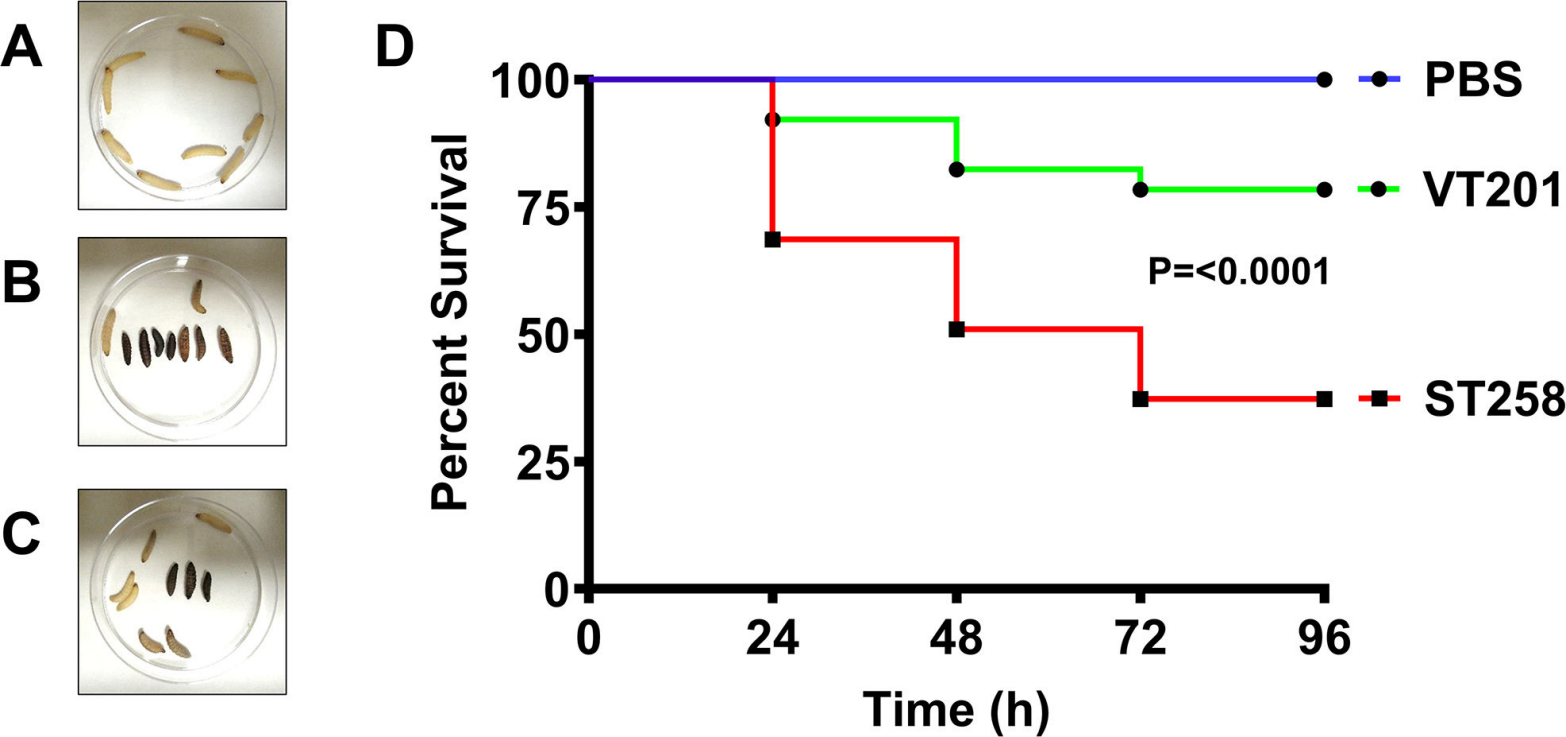
YqjA and YghB are required for virulence of *K. pneumoniae* in *Galleria mellonella* larvae. (**A**) Larva injected with sterile PBS pH 7.4 resulted in no death was used as the control. (**B**) Larvae injected with wild-type ST258 cell suspension made in PBS pH 7.4. (**C**) Larvae injected with VT201 cell suspension made in PBS pH 7.4. (**D**) Kaplan-Meier survival curve showing the killing rate of larvae was slower by VT201 compared to wild-type ST258. Each experiment was repeated three times.

We hypothesized VT201 will also show a decrease in virulence due to its altered levels of capsule. To measure virulence in an insect model, we injected PBS or 5.0 × 10^5^ wild-type ST258 or VT201 into *Galleria mellonella* larvae. We compared the survival rate every 24 hours for a duration of 4 days. At selected time points, we observed the survival percentage of larvae infected with VT201 was significantly higher than wild type, while larvae injected with PBS survived for the duration of the experiment (Fig. 10A through D). Based on these findings, we conclude VT201 is less virulent than wild type and highlights a requirement of *Klebsiella* YqjA and YghB for virulence.

## DISCUSSION

*Klebsiella pneumoniae* represents an important threat to human health as well as a source of antibiotic resistance. The DedA superfamily has been linked to both virulence and antibiotic resistance in several gram-negative organisms (16–18, 20). *E. coli* YqjA and YghB are together required for normal growth, cell division, and drug and biocide resistance (20, 21, 28), while *E. coli* YqjA by itself is required for growth at alkaline pH (15). We hypothesize that the bacterial DedA proteins function as proton-dependent transporters due to the presence of conserved membrane-embedded acidic and basic amino acids that are essential for their activity (20, 26). Such charged amino acids are a hallmark of proteins belonging to the major facilitator superfamily of membrane transporters which catalyze the transport of diverse substrates across the membrane in a proton-dependent manner (51). Mutation of *Klebsiella yqjA* and *yghB* affected growth, calcium uptake, alkaline tolerance and, when introduced into a virulent strain, capsule formation, resistance to phagocytosis, and virulence.

We used Percoll gradients to estimate the capsule formation and found a significant lack of capsule produced by VT201. Capsule is one of the major virulence factors in *Klebsiella’s* arsenal to resist phagocytosis by immune cells (12, 13, 49, 52, 53), and capsular mutants are more susceptible to phagocytic uptake (45). In this study, we observed a significantly higher number of VT201 phagocytosed by AMs, but post-phagocytotic survival is not impacted by the mutations, likely due to the presence of the PhoPQ two-component system and other signaling pathways activated in this environment (40). Capsule is assembled in *K. pneumoniae* and other bacteria using Und-P as a substrate creating Und-PP-linked hexoses (54). Recent studies reported the identification of DedA family proteins in a screen for potential recycling flippases of Und-P (46, 47), possibly explaining the impact upon capsule synthesis in this *Klebsiella* strain.

The wax moth *Galleria mellonella* and other invertebrate models of bacterial virulence are becoming increasingly common due to lower costs than rodent models and lack of ethical constraints (55). Insects possess an innate immune response that is similar to that of mammals (56, 57). We hypothesized VT201 would be compromised for virulence in the insect model due to lower capsule content. We observed the survival percentage of larvae infected with VT201 was significantly higher than wild type, consistent with what has been observed in this insect model using *K. pneumoniae* capsule mutants (58). These results highlight the requirement of *Klebsiella* YqjA and YghB for virulence.

While absence of capsule, sensitivity to phagocytosis, and decreased virulence may be linked to a loss of Und-P recycling, the sensitivity of VT101 to EDTA and SDS is far less obvious. The outer membrane (OM) in gram-negative bacteria is a structurally compact, selectively permeable hydrophobic barrier (54). Due to its strong hydrophobicity, the OM reduces the entry of toxic compounds such as antibiotics, bile salts, and detergents (59). The Mla (maintenance of lipid asymmetry) pathway is used to transport phospholipids between the inner membrane and OM of gram-negative bacteria (60–63), while the Bam pathway is needed for assembly of OM proteins (64). The sensitivity of VT101 toward EDTA and SDS resembles similar sensitivity of *E. coli* and *Acinetobacter baumannii* Δ*mla* mutants (61, 62) and the *E. coli* Δ*bamD* mutant (65), each of which displays defects in OM lipid asymmetry. Ca^2+^ and Mg^2+^ take part in electrostatic cross-links with negatively charged phosphate groups of LPS (33), and EDTA causes OM destabilization and permeabilization (34, 66–70). Further investigation is needed to understand if there is a link between the DedA family and phospholipid transport in *K. pneumoniae,* but sensitivity of VT101 toward SDS/EDTA may be a result of altered OM symmetry. In light of this, we note that a DedA protein from *Bacillus subtilis* has been shown to play a role in phosphatidylethanolamine redistribution across the cytoplasmic membrane (71).

The growth and alkaline pH sensitivity of VT101 at elevated temperature can be reversed by the addition of divalent cations (Ca^2+^ or Mg^2+^) to the growth medium. This indicates a possible alteration of divalent cation homeostasis in VT101. CorA, MgtE, and MgtA are the three distinct classes of Mg^2+^ transporters identified in bacteria (72–74). CorA and MgtE are considered primary Mg^2+^ transporters using membrane potential as energy source, while MgtA is a P-type ATPase (38, 75). In VT101, the growth sensitivity at elevated temperature might be a result of altered divalent cation homeostasis where CorA cannot function optimally due to the hyperpolarized membrane. Alternatively, DedA proteins may be a new class of divalent cation transporters. A recent study found that a DedA family protein plays a role in Mg^2+^ uptake and exopolysaccharide production by the plant pathogen *Xanthomonas campestris* (76).

To understand the role of the DedA proteins in divalent cation transport, Ca^2+^ uptake was measured. In prokaryotes, the intracellular [Ca^2+^] is tightly regulated and is maintained at nanomolar levels creating a concentration gradient against the external environment. Bacteria use ion channels (import Ca^2+^), Ca^2+^/H^+^ antiporters (efflux Ca^2+^), calcium-binding proteins, and ion condensation to maintain calcium homeostasis. In this study, direct measurement of Ca^2+^ uptake in VT101 showed an impairment in rapid uptake. The slower uptake of Ca^2+^ in the mutant could be due to the alteration of membrane potential which acts as a driving force for the operation of Ca^2+^ channels. The impairment of Ca^2+^ uptake in *K. pneumoniae* wild type using LaCl_3_, a Ca^2+^ channel inhibitor, was more drastic compared to the Ca^2+^ uptake profile of VT101. It indicates the existence of additional pathways in VT101 to import Ca^2+^. These findings suggested *K. pneumoniae* YqjA and YghB may play a role in Ca^2+^ uptake by regulating Ca^2+^ channel activity directly or indirectly. These results are also consistent with a study with *E. coli* showing increased influx of Ca^2+^ following membrane depolarization (77).

In this work, we characterized two *K. pneumoniae* mutants VT101 and VT201 deficient in two redundant DedA family proteins with 63% amino acid identity. We observed significantly higher phagocytosis of VT201, and the strain is significantly less virulent. We showed VT101 is sensitive to EDTA, SDS, elevated temperature, alkaline pH, and chemical agents alkalizing cytoplasm. Overexpression of *K. pneumoniae yqjA* or *yghB* from an inducible plasmid complements each phenotype of VT101, illustrating the functional redundancy between these proteins. Addition of divalent cations (Ca^2+^ or Mg^2+^) alleviated the sensitivity of VT101 toward each of these growth conditions. Divalent cations likely exert their effect by reversal of the membrane hyperpolarization (18). Based on these results, we conclude VT101 struggles to maintain divalent cation homeostasis due to changes in membrane polarization, while the loss of virulence of VT201 is linked to defects in capsule synthesis and subsequent resistance to phagocytosis.

## MATERIALS AND METHODS

### Media, plasmids, bacterial strains, and chemicals

Table 2 lists the bacterial strains and plasmids used in this study. Bacterial strains were typically grown in Luria-Bertani medium (1% NaCl, 1% tryptone, 0.5% yeast extract) at pH 7.0 unless otherwise indicated. For lambda red recombination, *K. pneumoniae* was grown in low-salt LB (LSLB) (0.5% NaCl, 1% tryptone, 0.5% yeast extract). Biofilm minimal medium 8 (BMM8) was used to grow *K. pneumoniae* for monitoring intracellular Ca^2+^ (78). BMM8 consists of 9.0 mM sodium glutamate, 50 mM glycerol, 0.08 mM MgSO_4_, 0.15 mM NaH_2_PO_4_, 0.34 mM K_2_HPO_4_, 145 mM NaCl, 200 µL trace metals, and 1 mL vitamin solution (per liter of medium). The trace metals mixture was prepared with 5.0 g CuSO_4_·5H_2_O, 5.0 g ZnSO_4_·7H_2_O, 5.0 g FeSO_4_·7H_2_O, and 2.0 g MnCl_2_·4H_2_O per liter of 0.83 M concentrated hydrochloric acid. The vitamin solution was prepared by dissolving 0.5 g thiamine and 1 mg biotin (per liter of the final medium). Filter-sterilized trace metals, MgSO_4_, and vitamin solution were added aseptically to autoclaved medium adjusted to a pH of 7. Antibiotics used in this study were purchased from Sigma-Aldrich and used at the following concentrations: apramycin (Apr) 50 µg/mL, tetracycline (Tet) 12.5 µg/mL, hygromycin (Hyg) 100 µg/mL, and chloramphenicol (Cam) 100 µg/mL. Ethylenediaminetetraacetic acid, calcium chloride dihydrate (CaCl_2_·2H_2_O), and magnesium sulphate heptahydrate (MgSO_4_·7H_2_0) were purchased from Sigma-Aldrich. JC-1 dye was purchased from ThermoFisher. *Galleria mellonella* larvae were purchased from Carolina Biological Supply. Primers were purchased from Sigma-Aldrich (Table S1).

**TABLE 2:** Bacterial strains and plasmids

Strain or plasmid	Description	Source or reference
**Strains**		
*Escherichia coli*		
W3110	Wild-type *Escherichia coli*, F-,λ-,IN (*rrnDrrnE)1, rph-1*	*E. coli* genetic stock center, Yale University
BC202	W3110 ∆*yqjA*::Tet^R^ ∆*yghB781::Kan^R^*	(28)
XL1 Blue	*recA*1 *endA1 gyrA96 thi-1 hsdR17 supE44 relA1 lac [F´ proAB lacIqZΔM15 Tn10* (Tet^R^)	Stratagene
SM10	*thi thr leu tonA lacY supE recA::RP4-2-Tc::Mu Km λpir*	(79)
*Klebsiella pneumoniae*		
MKP103	Carbapenemase (KPC-3) deletion derivative of *K. pneumoniae* KPNIH1	*K. pneumoniae* TN library, University of Washington (80)
*yghB*::Tn30	Tn30 Cam^r^ insertion at 482nd position of *K. pneumoniae yghB* in wild-type MKP103	
VT101	*K. pneumoniae* MKP103; Δ*yqjA*, Δ*yghB*	This study
ST258	MDR *K. pneumoniae* ST258 (RH201207)	(81)
VT201	*K. pneumoniae* ST258; Δ*yqjA* Δ*yghB*	This study
		
**Plasmids**		
pBADApr^R^	Expression vector; *araBAD* promoter, Apr^R^	(18)
pBAD (KpYqjA)	pBAD expressing *K. pneumoniae yqjA* with N-terminal His_6_ tag	This study
pBAD (KpYghB)	pBAD expressing *K. pneumoniae yghB* with N-terminal His_6_ tag	This study
pSCrhaB2	Expression vector; oripBBR1*rhaR, rhaS, P*rhaBTetR *mob*	(82)
pSC (KpYqjA)	pSCrahB2 expressing *yqjA*	This study
pSC (KpYghB)	pSCrahB2 expressing *yghB*	This study
pACBSR-Hyg^R^	Arabinose-inducible vector consisting of λ-Red recombinase gene with hygromycin resistance cassette	(24)
pIJ773	Template used to amplify Apr^R^	(24)
pFLP Tet	Rham-inducible flp, TS ori	(83)
pCRE3	Ap^r^ Zeo^r^; contains *rhaS-rhaR-P_rhaBAD_-cre* from pPS2205, *ble-ori*_1600_-*rep* (Ts*_Bt_*) from pPS2165, and *oriT-ori* from pFLP2	(84)

### Deletion of transposon Tn30 marker by Cre/lox recombination

Excision of the Cam^r^ transposon marker was carried out as described (84). Tn30 has directly repeated *loxP* sequence at the end of the transposon on both ends (Fig. S4A through D). Recombination was carried out by inducing Cre recombinase in non-replicating plasmid pCRE3 (Table 2). *K. pneumoniae yghB*::Tn30 has an insertion of the transposon marker at the 482nd position of *yghB*. The strain was electroporated with 100 ng of pCRE3 and selected with 500 µg/mL Zeocin. Eight positive transformants with pCRE3 were randomly selected and streaked on LB plates containing 0.2% rhamnose and 500 µg/mL Zeocin. Colonies were screened for sensitivity to 100 µg/mL Cam. Colonies showing cam^s^ indicated the loss of Cam^r^ cassette. After the excision of transposon marker, pCRE3 plasmid was cured by streaking a single colony daily onto a fresh LB for a period of 15 days and testing for sensitivity to 500 µg/mL Zeocin. PCR amplification was performed to confirm the excision of transposon marker (Fig. S4).

### Deletion of *yqjA* and *yghB* from *K. pneumoniae*

Genetic deletion of *yqjA* and *yghB* was carried out using λ red recombination (24, 85). *yqjA* was deleted from *K. pneumoniae* MKP103 in the *yghB*::Tn30 background (library strain KP09345). Both *yqjA* and *yghB* were deleted in virulent strain *K. pneumoniae* ST258 and grown in LSLB. pACBSR (Hyg^r^) was electroporated at 30°C, and transformants were selected on Hyg 100 µg/mL. PCR amplification, using Q5 DNA polymerase (New England Biolabs), was carried out using the knockout primers (Table S1) and pIJ773 Apr^r^ plasmid (24) as the template. The PCR product was a linear DNA fragment having 60 bp overlap with the target gene on both 5′ end and 3′ end. Linear DNA was incubated overnight with *DpnI* at 37°C and purified. Strains harboring pACBSR were grown with 0.1% arabinose to mid-exponential phase at 30°C. Cells were washed 3× with 10% glycerol and resuspended in 250 µL 10% glycerol. Linear DNA (800–1,000 ng) was mixed with 50 µL of the cell suspension and electroporated. The electroporated sample was mixed with 1 mL SOC medium, and following outgrowth without selection for 1 hour, cells were selected with 50 µg/mL Apr at 37°C for 24 hours. Colonies were screened by PCR using four nested primers, P1F, P2R, P3F, and P4R (Table S1). A rhamnose-inducible pFLP plasmid (83) was used to excise the apramycin resistance cassette leaving a flippase recognition target (FRT) scar. Plasmid was subsequently cured by growth in the absence of selection on LB plates at 37°C. Genome sequencing was carried out to confirm the absence of unintended mutations. Phenotypes of both VT101 and VT201 were complemented by plasmid expression of *yqjA* and *yghB*. Confirmation of all deletion mutants can be found in the supporting information (Fig. S4 to S7).

### Cloning of *K. pneumoniae yqjA* and *yghB*

*K. pneumoniae* MKP103 genomic DNA was extracted using Easy-DNA Kit (Invitrogen). PCR amplification was performed with primers KpyqjA1 and KpyqjA2 for cloning *Klebsiella* YqjA and primers KpyghB1 and KpyghB2 for cloning *Klebsiella* YghB (Table S1). The DNA fragments were purified and digested with *KpnI* and *HindIII* (New England Biolabs). pBAD Apr^r^ plasmid was similarly digested and dephosphorylated. A 20-µL ligation reaction using Hi-T4 DNA ligase (New England Biolabs) was incubated at 25°C for 3 hours, then transformed into SM10 (λ pir) (86) competent cells. Positive transformants were selected on 50 µg/mL Apr.

### Measurement of membrane potential

Membrane potential (ΔΨ) was measured using JC-1 dye using a previously published protocol (14).

### Measurement of Ca^2+^ uptake: construction of aequorin reporter plasmid

The aequorin gene was amplified from pMMB66EH (courtesy of Dr. Delfina Dominguez; University of Texas at El Paso) (87) by using AeqF and AeqR primers (Table S1). The DNA fragment was digested with *KpnI* and *EcoRI* (New England Biolabs). pBAD Apr^r^ plasmid was similarly digested and dephosphorylated. A 20-µL ligation reaction was incubated at 25°C for 3 hours, then transformed into SM10 (λ pir) competent cells. Transformants were selected with 50 µg/mL Apr.

### Measurement of Ca^2+^ uptake: monitoring free intracellular Ca^2+^

To monitor the intracellular free Ca^2+^ in wild-type *K. pneumoniae* and VT101, we followed previously published protocol with several modifications (43). The cultures were grown shaking in BMM8 for 8 hours at 30°C, normalized to an OD_600_ of 0.25, and used to inoculate (1%) 100 mL of BMM8. After allowing wild-type and VT101 cultures to grow until early-log phase (12 and 16 hours, respectively), 0.5% arabinose was added to induce (apo)aequorin expression. After an additional 8 hours of incubation, 25 mL of each culture was collected and provided with 5 µM (final) coelenterazine to reconstitute aequorin. Following the procedure described in reference (43), the coelenterazine-treated cells were normalized to an OD_600_ of 0.4, aliquoted (100 µL) into a 96-well plate, and luminescence was recorded using a Synergy Mx plate reader. Basal luminescence was recorded for 1 min every 5 s followed by injecting 1 mM (final) CaCl_2_ prepared in 25 mM HEPES buffer. The samples were mixed for 1 s, and luminescence was recorded for 20 min every 5 s. As a negative control, 25 mM HEPES buffer was injected alone, following which no significant fluctuations in luminescence were recorded. To ensure a sufficient amount of aequorin was available for binding Ca^2+^, following each experiment, the remaining aequorin was evaluated by treating samples with a discharge buffer containing 2% Nonidet 40, to permeabilize the cell, along with 12.5 mM CaCl_2_. This would permeabilize the membranes and let an excess of Ca^2+^ enter the cells and interact with aequorin. Luminescence was then recorded for an additional 2 min every 5 s. To ensure the increase in luminescence was specific to Ca^2+^, both MKP103 and VT101 cells were treated for 10 min with 5 µM calcimycin, a calcium ionophore, followed by CaCl_2_ injection and measuring luminescence. Calcimycin was dissolved in 3% DMSO with 10 µg/mL of compound 48/80, known to permeabilize the OM (88). We also included a negative control composed of 10 µg/mL compound 48/80 in 3% DMSO. Upon Ca^2+^ injection, calcimycin-treated MKP103 and VT101 cells showed a rapid spike of intracellular Ca^2+^ at 68 s (Fig. S8A and B), whereas DMSO treatment showed no impact when compared to non-treated cells. Finally, to test the involvement of Ca^2+^ channel activity in *K. pneumoniae* Ca^2+^ uptake, we treated MKP103 and VT101 cells with 600 µM LaCl_3_, a Ca^2+^ channel antagonist (89). For this, the inhibitor was added to the samples and incubated for 10 min in the dark at room temperature without shaking followed by monitoring luminescence as described above.

### Percoll gradient assessment of capsule

The Percoll (GE healthcare) gradient was produced as described previously (48).

### Measurement of phagocytosis by murine alveolar macrophages

The phagocytosis assay was performed using a previously published protocol (90). Murine alveolar macrophage cells (MH-S cells, ATCC: CRL-2019) grown with in RPMI 1640/10% FBS media with L-glutamine were normalized to a concentration of 4 × 10^5^ cells/mL of culture media. The cells were seeded in CELLSTAR 24-well plate (Greiner Bio-One) at the concentration of 2 × 10^5^ cells per well without antibiotic. The cells were incubated overnight in an incubator at 37°C supplied with 5% CO_2_. MH-S cells were infected with multiplicity of infection of 10 and kept at 37°C, 5% CO_2_. Infected AMs were incubated for 1 hour and 30 min for phagocytosis to occur and then were washed twice with modified DPBS to wash extracellular bacteria and supplied with fresh culture media with 300 µg/mL gentamicin (Quality Biological) to kill extracellular bacteria. To perform only the gentamicin wash control experiment (Fig. S3), the media containing gentamicin were removed and replaced with fresh media. Then fresh media were changed every hour until the 6-hour time point. At 2, 6, and 24 hours post infection, the corresponding wells were washed once with DPBS, and the cell lysate was collected from each well in 1 mL of 0.1% Triton X-100 in deionized water. The cell lysate was serially diluted 10^−1^ twice, and 50 µL of each dilution was plated in LB agar plates and incubated at 30°C to estimate the number of intracellular bacteria. The strains were compared for phagocytosis and killing at different time points.

### Virulence measurement using *Galleria mellonella*

Overnight cultures of indicated strains were prepared and diluted to 1 × 10^8^ CFU/mL. Larvae were weighed individually and distributed uniformly between three biological replicates. The weight of larvae ranged from 200 to 320 mg. Each biological replicate had six individual larvae in a sterile petri dish. The left proleg of each larva was surface sterilized with a sterile cotton swab dipped in 70% ethanol before injection. Five microliters of cell suspension (5 × 10^5^ CFU) or PBS, pH 7.4 were drawn using Hamilton syringe (catalog no. 80300) to inject into the larvae. After injection, larvae were returned to their respective petri dish and incubated at 30°C. Survival of larvae was monitored every 24 hours over a period of 96 hours.

### Susceptibility assays

MIC values were determined by broth microdilution. Sensitivity assays on LB agar plates were performed by spotting 5 µL of serially log_10_-diluted bacterial cells from overnight culture onto plates and incubated as indicated in the figure legends.

### Statistical analysis

Each data point represents mean ± standard error mean. Each experiment was repeated three times with nine independent biological replicates. Data analysis was carried out using the unpaired Mann-Whitney test to determine statistical significance.

## Data Availability

Upon request, the data that support the findings of this study are available from the corresponding author.

## SUPPLEMENTAL MATERIAL

Supplemental material spectrum.03807-23-s0001.pdf Table S1 and Figures S1 to S8.

ASM does not own the copyrights to Supplemental Material that may be linked to, or accessed through, an article. The authors have granted ASM a non-exclusive, world-wide license to publish the Supplemental Material files. Please contact the corresponding author directly for reuse.
