# Supplementary material for: Klebsiella pneumoniae DedA family proteins have redundant roles in divalent cation homeostasis and resistance to phagocytosis
Source: Microbiol Spectr. 2024 Jan 12;12(2):e03807-23. doi: 10.1128/spectrum.03807-23 (PMC10846249; doi:10.1128/spectrum.03807-23)
Supplement: Supplemental material — Table S1 and Figures S1 to S8. [file spectrum.03807-23-s0001.pdf]

Supplemental material:

Klebsiella pneumoniae DedA family proteins have redundant roles in divalent cation homeostasis and resistance to phagocytosis.

Vijay Tiwari[1*], Amit Sharma[2], Reygan Braga[3], Emily Garcia[1], Ridhwana Appiah[4], Renee Fleeman[4], Basel H. Abuaita[2], Marianna Patrauchan[3] and William T. Doerrler[1#]

Affiliations:

[1] Department of Biological Sciences, Louisiana State University, Baton Rouge, LA, USA

[2] Department of Pathobiological Sciences, LSU School of Veterinary Medicine, Louisiana State University, Baton Rouge, LA, USA

[3] Department of Microbiology and Molecular Genetics, College of Arts and Science, Oklahoma State University, Stillwater, OK, USA

[4] Burnett School of Biomedical Sciences, College of Medicine, University of Central Florida, Orlando, FL, USA

* Current address: Department of Pharmacology, University of Nevada, Reno School of Medicine, Reno, NV

# Correspondence: William T. Doerrler wdoerr@lsu.edu

Table S1. Oligonucleotide primers used in this study.

Primer name	Primer Sequence
KpyqjA1	GAAC**GGTACC**ATGGAACTATTTACCCAGTTATTGAATGCCTTGTGGG
KpyqjA2	GTTC**AAGCTT**TTAGCCCCGGCTTCTGTATTTTTTCTTCC
KpyghB1	GAACGGTACCATGGTTGTCATTCAAGAGATTGTCGCCG
KpyghB2	GTTCAAGCTTTCAGGCGCTGCAATATTTCTTTTTAATGACCAC
Tn30	CTGCGAAGTGATCTTCCGTCAC
ST258_*yqjA*_KO1	TCGCCCTATATGCCACGCGCTCAGACGTGCCGTTAACCGATGTACCAGGAATCGTGAATGATTCCGGGGATCCGTCGACC
ST258_*yqjA*_KO2	GAGAACCACCCAGCAGCCAGCTGAACTGGCGCAGGGAAGGGCGTTTCATGACCATCATTATGTAGGCTGGAGCTGCTTC
P1F_*yqjA1*	ATTAAT**AAGCTT**GAGATGATGCCAGACGGAGAAACC
P1F_*yqjA2*	AATATT**GAATTC**ACAGTGAGGCTGGAGTAAGCAAGTCATATATAG
P2R_*yqjA*	CATTCTCGAGATAATCGACGCGTACCAAC
P3F_*yqjA*	GTTGGTACGCGTCGATTATCTCGAGAATG
P4R_*yqjA1*	AATATT**GAATTC**GCGTTCAGAGCGCCTAAGCAG
P4R_*yqjA2*	ATTAAT**AAGCTT**GAGTTATCGTCCTCCAGAAGGGCAATAATATAACC
KpyqjA1b	ATTATT**CATATG**ATGGAACTATTTACCCAGTTATTGAATGCC
KpyqjA2b	AATATT**TCTAGA**TTAGCCCCGGCTTCTGTATTTTTTC
KpyghB1b	AATATT**GAATTC**GACAATTTCAGCCTGTTCAGTATGGCCTATTC
KpyghB2b	ATTAAT**AAGCTT**CAGCGTTTCACCATAATTTTAAAGCGGTGAC
ST258_*yghB*_KO1	CATGGCCGGGATTTCTGGCCTGCCGAACCGCCGCTTCCAGTTCTTCAACTGGCTGAGCGCCGAATAAGGGACAGTGAAGAAGGAACACC
ST258_*yghB*_KO2	ACAAACGGGATCATGCTCAGCGCATAGCCAAGACCCGTCACCACACAGACCCACAGCAGGCTGCAGGAATTCGATGTGTAGGCTG
P1F_*yghB*	CACATTGGTCTCGAAAATGTTGACGATCTG
P2R_*yghB*	CCTGCCAAATGTAAAGCGCAGC
P3F_*yghB*	CGCTGCGCTTTACATTTGGCAG
P4R_*yghB*	GAGTTTAAGCGCTACTTCGGTTTGAC
KpyqjA1	ATTATT**CATATG**ATGGAACTATTTACCCAGTTATTGAATGCC
KpyqjA2	AATATT**TCTAGA**TTAGCCCCGGCTTCTGTATTTTTTC
KpyghB1	ATTATT**CATATG**ATGGTTGTCATTCAAGAGATTGTCGCC
KpyghB2	AATATT**TCTAGA**TTAGGCGCTGCAATATTTCTTTTTAATGACCACG
AeqF	ATTAAT**GGTACC**AAATGACCAGCGAACAATACTCAGTC
AeqR	ATAATT**GAATTC**CAGAGTTTCTTAGGGGACAGCTC

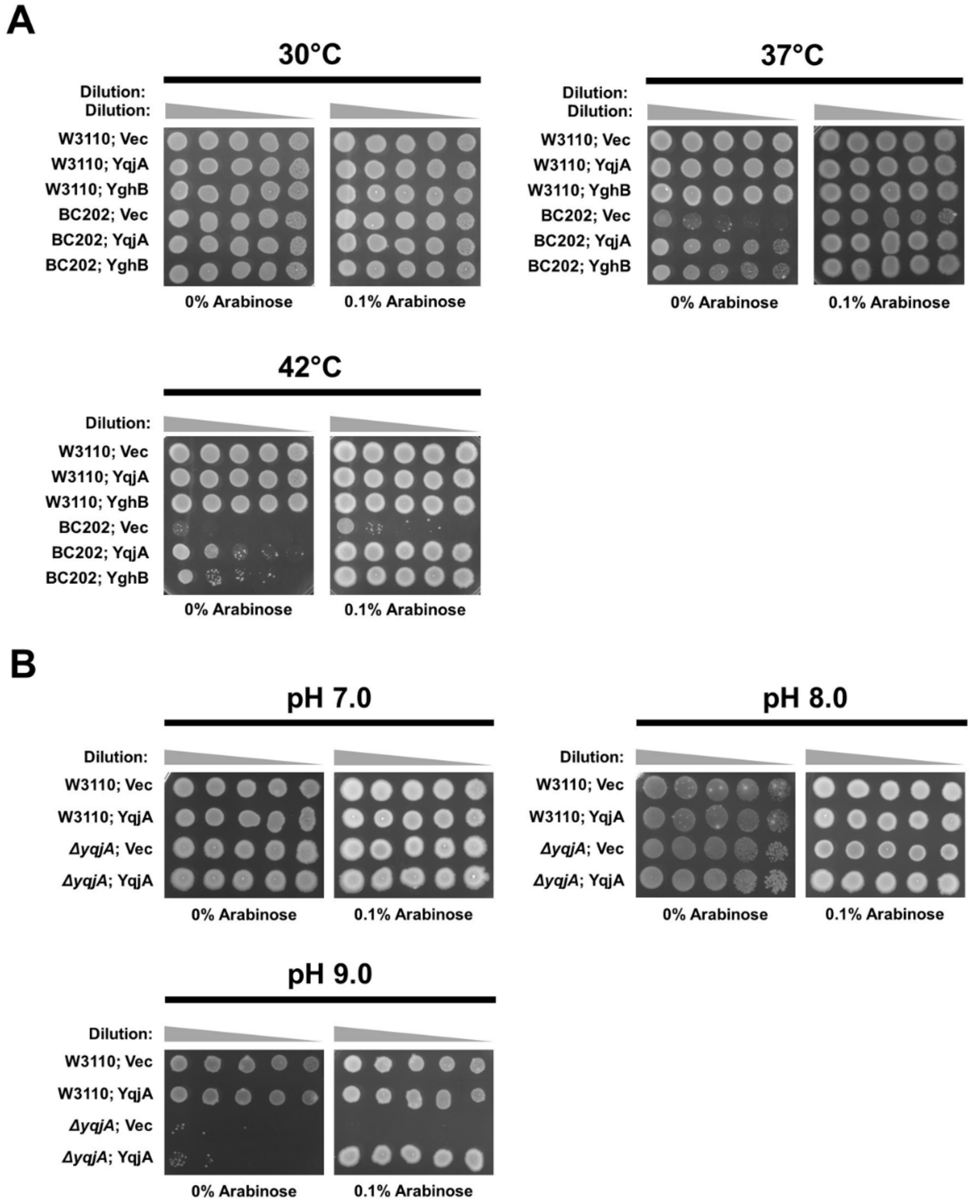

Fig. S1. Complementation of growth defects of *E. coli dedA* family mutants by expression of *Klebsiella yqjA* or *yghB*. (A) Restoration of growth of *E. coli* BC202 (Δ*yqjA*, Δ*yghB*) at elevated temperature. Plates were incubated at indicated temperatures for 24 h. (B) Restoration of growth of *E. coli* Δ*yqjA* at alkaline pH. Plates were incubated at 37°C for 36 h. 1:10 dilutions of overnight cultures of indicated strains were spotted on LB plates with 0 or 0.1% arabinose. See Table 2 for description of strains and plasmids. Experiments were repeated three times.

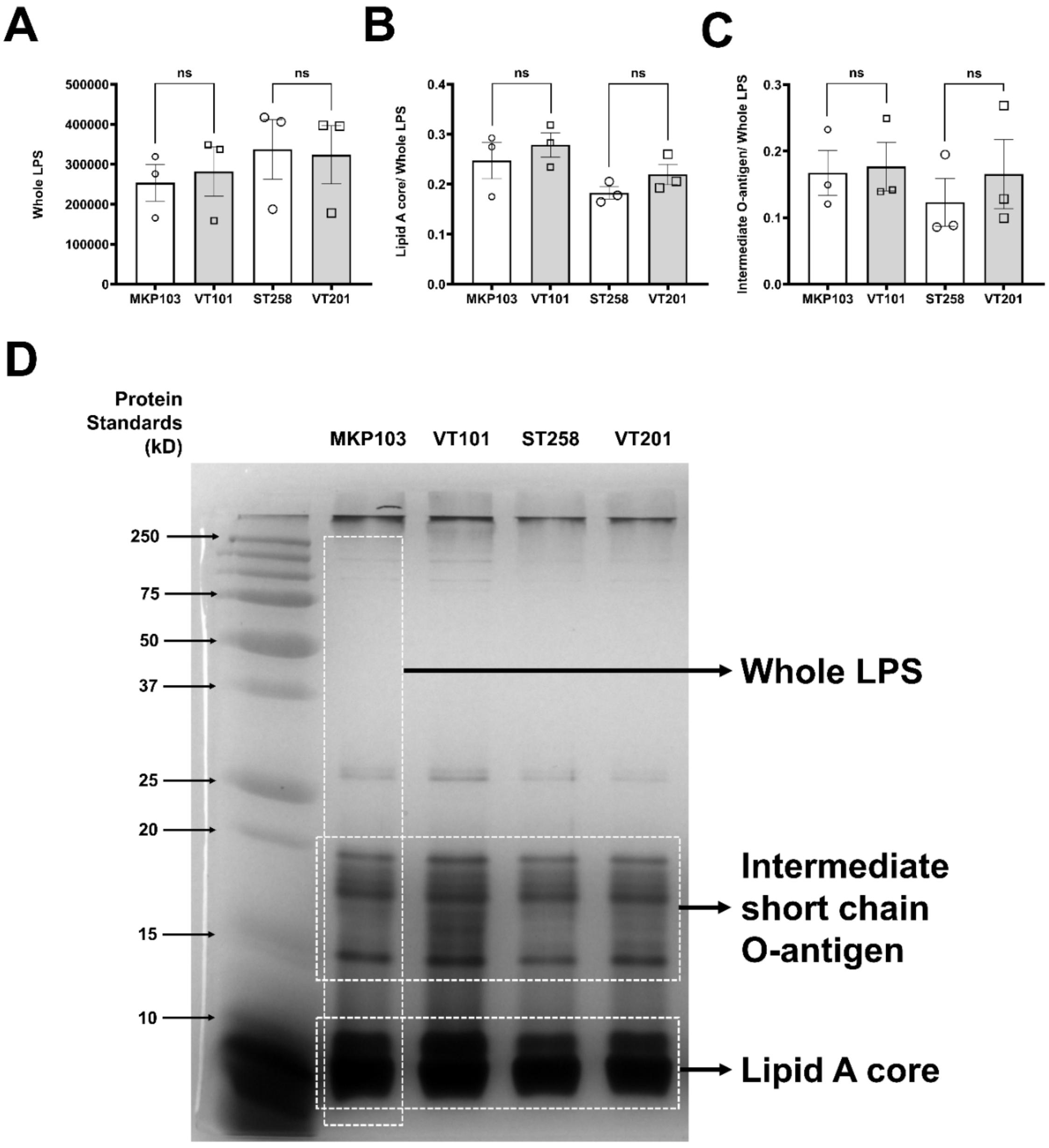

Fig. S2. LPS profile of *K. pneumoniae* strains. Quantification of (A) whole LPS, (B) LPS core/whole LPS ratio, and (C) intermediate O-antigen/whole LPS ratio of LPS samples from *K. pneumoniae* MKP103, VT101, *K. pneumoniae* ST258 and VT201. (D) Representative image of silver staining experiment. Experiment was repeated three times. Bars represent mean ± SEM of three biological replicates. The Mann-Whitney test was used to perform statistical analysis. Statistical comparisons were considered significant at $p<0.05$. ns: non-significant.

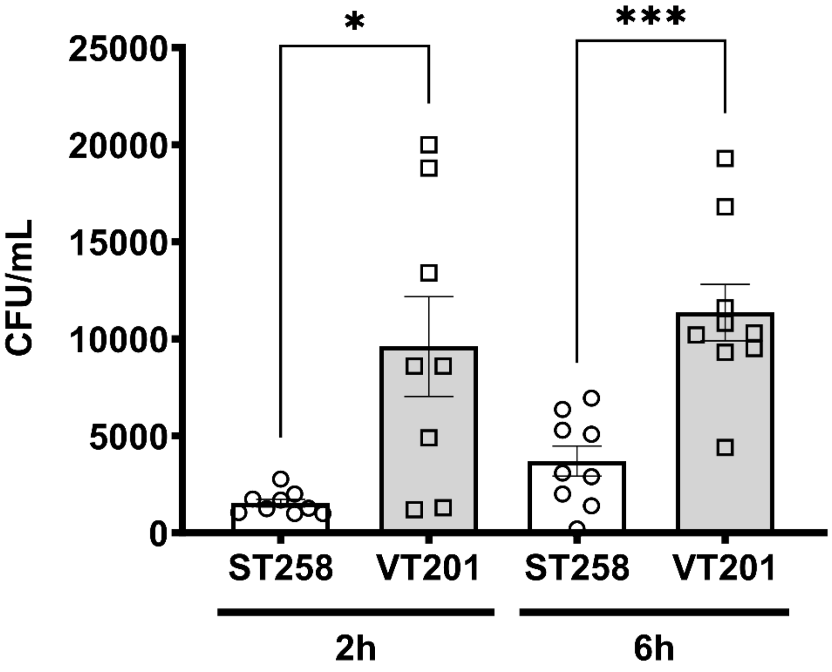

Fig. S3. Macrophage killing assay with short term gentamicin exposure. Because V201 was found unexpectedly to be more resistant to gentamicin than ST258 (Table 1), the phagocytosis rate of wild type *K. pneumoniae* and VT201 by alveolar macrophages was estimated using short term gentamicin exposure (30 mins) followed by subsequent hourly washing using fresh culture media without gentamicin to remove extracellular bacteria. Bars represent mean ± SEM of nine biological replicates. The experiment was repeated three times. The Mann-Whitney test was used to perform statistical analysis. Statistical comparisons were considered significant at $p<0.05$. *: $p < 0.05$. ***: $p < 0.001$.

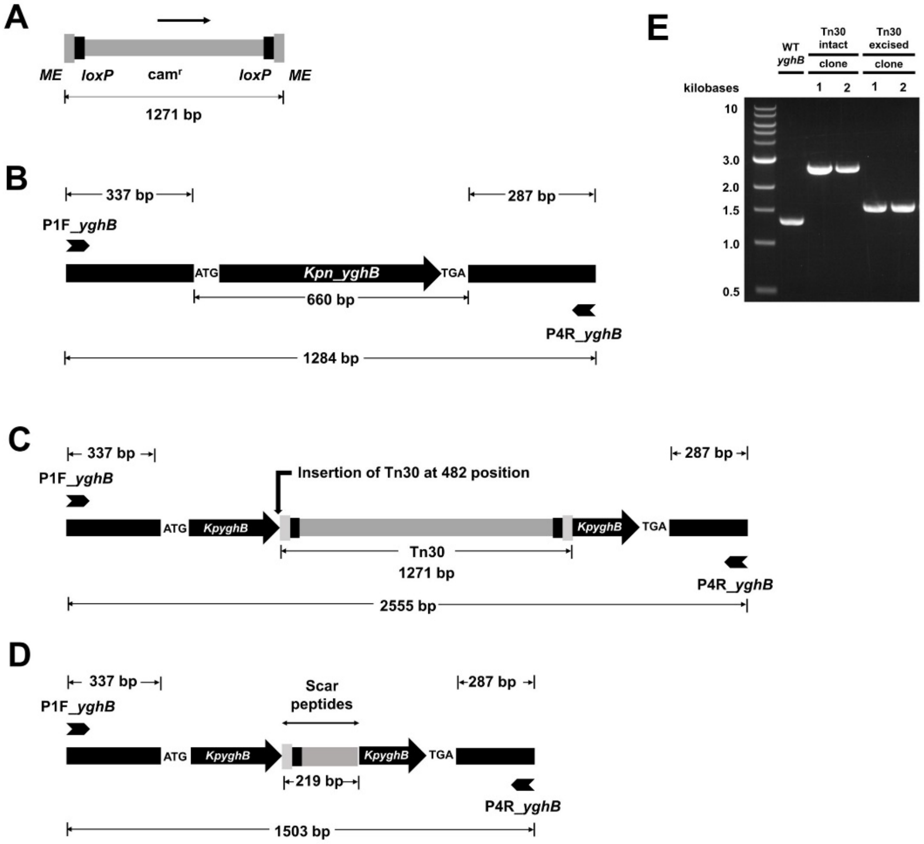

Fig. S4. Deletion of Tn30 from *Klebsiella yghB*::Tn30 by Cre/*lox* recombination. (A) Overall structure of transposon Tn30. The length of Tn30 is 1271 bp with mosaic ends (ME) and *loxP* recombination sites flanking the chloramphenicol resistance marker (cam^r). (B) Genomic map of *Klebsiella yghB*. (C) *Klebsiella yghB* deletion using Tn30 mutagenesis. MKP103 was mutagenized using Tn30-tranposase complex and selection of mutant colonies were made with chloramphenicol. *Klebsiella yghB* was mutagenized with Tn30 insertions at three different positions. *Klebsiella yghB*:: Tn30 (library strain KP09345) was obtained from the *K. pneumoniae* mutant library at The University of Washington (UW) (1). (D) Deletion of Tn30 in *Klebsiella yghB*:: Tn30 by Cre/*lox* recombination. The *loxP* x *loxP* recombination event will excise the cam^r resistant marker leaving behind a scar peptide of 219 bp length (1). (E) Gel electrophoresis to confirm the excision of Tn30 in *Klebsiella yghB*. PCR products using primer sets, P1F_*yghB* & P4R_*yghB* was used to confirm the excision of Tn30. The PCR amplification will form PCR products of 1503 bp. PCR amplified wild type *yghB* was used as the control.

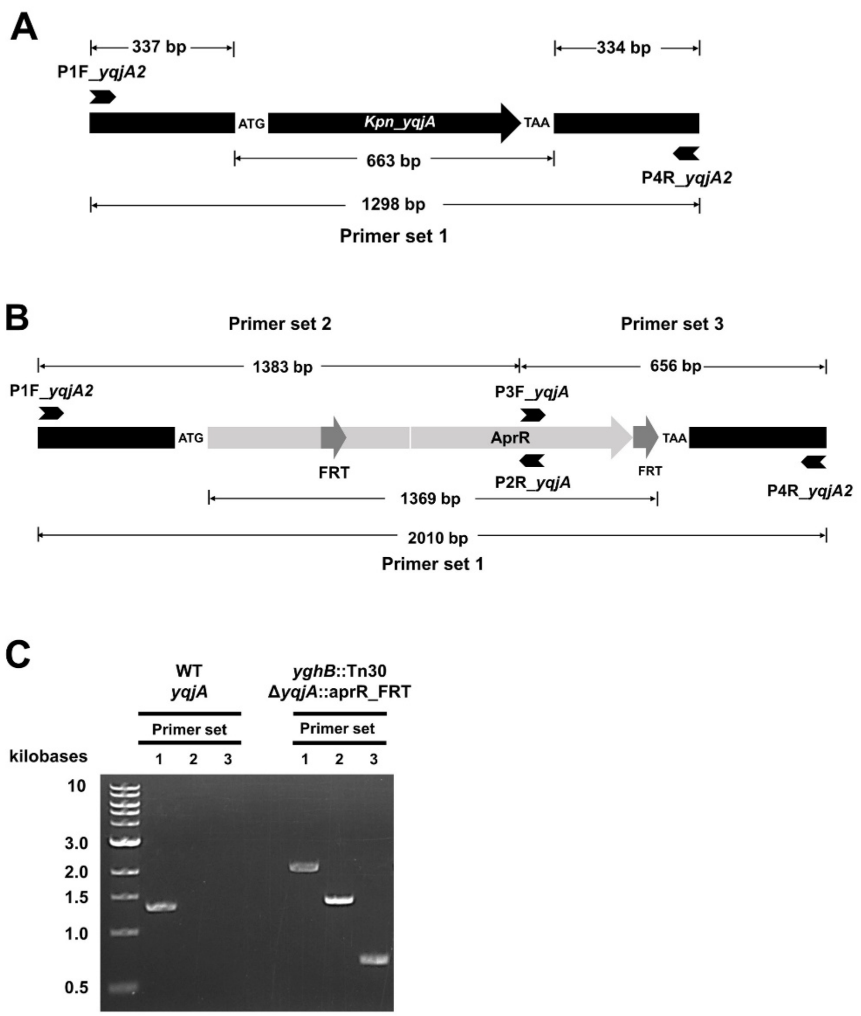

Fig. S5. Deletion of *Klebsiella yqjA* from *K. pneumoniae* MKP103. (A) Genomic map of *Klebsiella yqjA*. Length of *Klebsiella yqjA* is 663 bp. Primers used for the verification are designed upstream and downstream of the wild type (WT) *Klebsiella yqjA* forming PCR product size of 1298 bp length. (B) *Klebsiella yqjA* deletion map. Apramycin resistant (Apr^R) knockout cassette with flanking 60 bp homologous region from 5' and 3' end of *Klebsiella yqjA* was used to carry out homologous recombination. The knockout cassette replacing *Klebsiella yqjA* consisting of two FRT sites. The Apr^R cassette was excised using pFLP tetracycline resistant plasmid forming a clean Δ*yqjA* mutant. (C) Gel electrophoresis to confirm *Klebsiella yqjA* deletion. PCR products using three primer sets, P1F_*yqjA2* & P4R_*yqjA2* (primer set-1), P1F_*yqjA2* & P2R_*yqjA* (primer set-2) and P3F_*yqjA* & P4R_*yqjA2* (primer set-3) were used to confirm the deletion of *Klebsiella yqjA*. The PCR amplification using primer set 1, 2 and 3 will form PCR products of 2010 bp, 1383 bp and 656 bp sizes respectively. Wild type *yqjA* amplified with all three primer sets was used as the control. Primer set 1 will form a product of 1298 bp with WT *yqjA* but will not produce a PCR product with primer set 2 & 3.

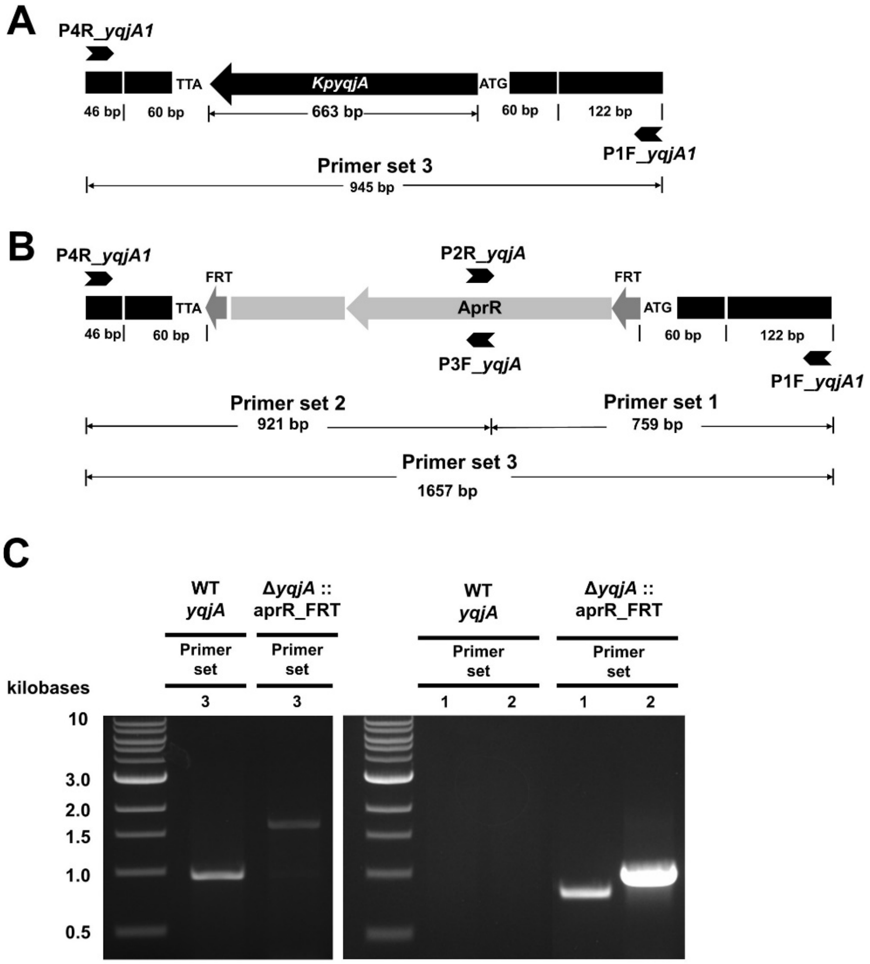

Fig. S6. Deletion of *Klebsiella yqjA* from *K. pneumoniae* ST258. (A) Genomic map of *Klebsiella yqjA*. Primers used for verification are designed upstream and downstream of the wild type (WT) *Klebsiella yqjA* forming PCR product size of 945 bp length. (B) *Klebsiella yqjA* deletion map. Apramycin resistant (Apr^R) knockout cassette with flanking 60 bp homologous region from 5' and 3' end of *Klebsiella yqjA* was used to carry out homologous recombination. The knockout cassette replacing *Klebsiella yqjA* consisting of two FRT sites. The Apr^R cassette was excised using pFLP tetracycline resistant plasmid resulting in clean Δ*yqjA*. (C) Gel electrophoresis to confirm *Klebsiella yqjA* deletion. PCR products using three primer sets, P1F_*yqjA1* & P2R_*yqjA* (primer set-1), P3F_*yqjA* & P4R_*yqjA1* (primer set-2) and P1F_*yqjA1* & P4R_*yqjA1* (primer set-3) were used to confirm the deletion of *Klebsiella yqjA*. The PCR amplification using primer set 1, 2 and 3 will form PCR products of 759 bp, 921 bp and 1657 bp sizes respectively. WT *yqjA* amplified with all three primer sets was used as the control. Primer set 1 & set 2 will not produce a PCR product and primer set 3 will result in a product of 945 bp length.

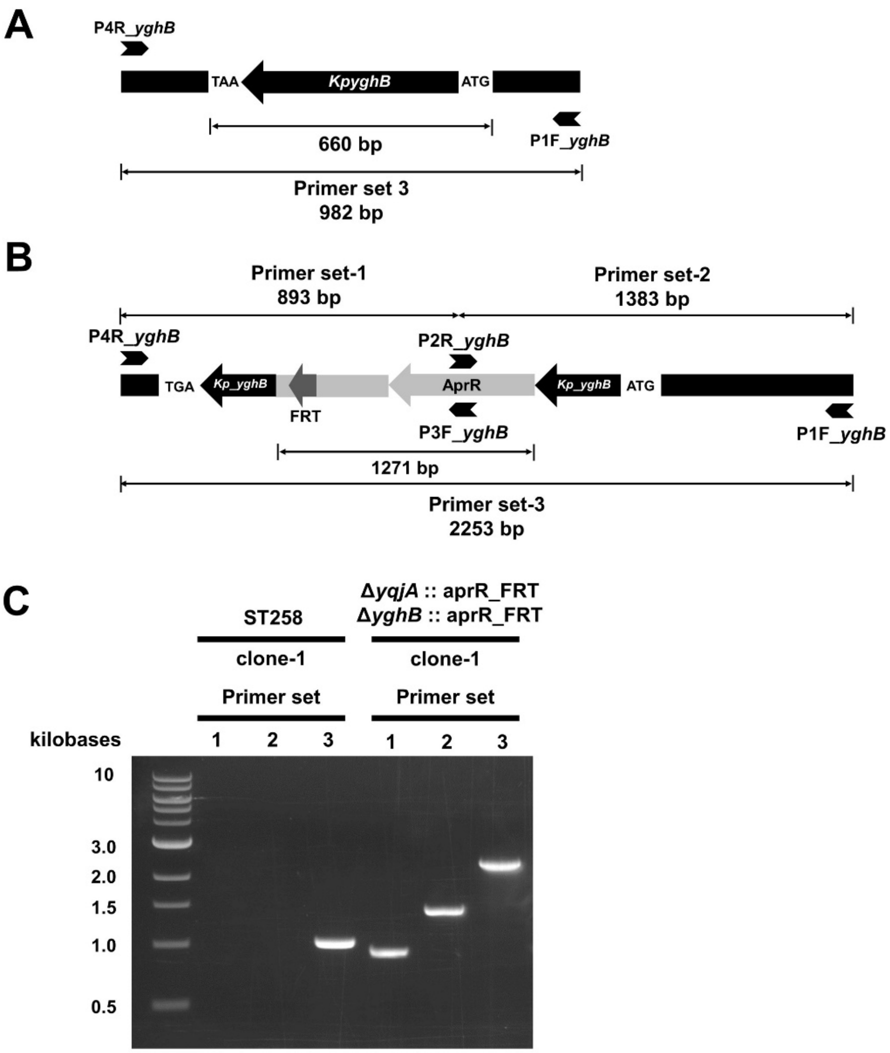

Fig. S7. Deletion of *Klebsiella yghB* from *K. pneumoniae* ST258. (A) Genomic map of *Klebsiella yghB*. Primers used for verification are designed upstream and downstream of wild type (WT) *Klebsiella yghB* forming PCR product size of 982 bp length. (B) *Klebsiella yghB* deletion map. Apramycin resistant (Apr^{R}) knockout cassette of 1271 bp length flanked by 60 bp homologous region from 5' and 3' end of *Klebsiella yghB* was used to carry out homologous recombination. The knockout cassette consists of only one FRT site so it cannot be excised rendering the mutant resistant to apramycin. (C) Gel electrophoresis to confirm *Klebsiella yghB* deletion. PCR products using three primer sets, P3F_*yghB* & P4R_*yghB* (primer set-1), P1F_*yghB* & P2R_*yghB* (primer set-2) and P1F_*yghB* & P4R_*yghB* (primer set-3) were used to confirm the deletion of *Klebsiella yghB*. The PCR amplification using primer set 1, 2 and 3 will form PCR products of 893 bp, 1383 bp, and 2253 bp sizes respectively. WT *yghB* gene amplified with all three primer sets was used as the control. Primer set 1 & set 2 will not produce a PCR product but primer set 3 will result in a product of 982 bp length.

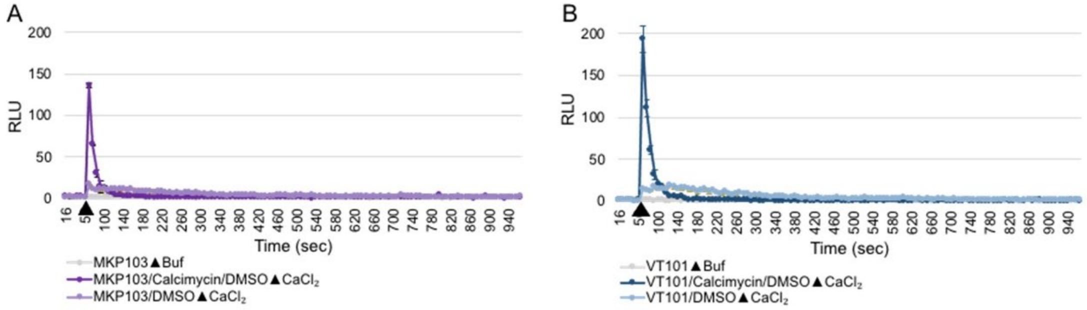

Fig. S8. Calcium uptake in *K. pneumoniae* was validated by treating cells with calcimycin, a calcium ionophore. (**A**) MKP103 and (**B**) VT101 were treated with 5 μM calcimycin before challenging the cells with 1mM $CaCl_2$.

References

1. Ramage B, Erolin R, Held K, Gasper J, Weiss E, Brittnacher M, Gallagher L, Manoil C. 2017. Comprehensive Arrayed Transposon Mutant Library of Klebsiella pneumoniae Outbreak Strain KPNIH1. J Bacteriol 199.
